# Combined Pharmacophore and Grid-Independent Molecular Descriptors (GRIND) Analysis to Probe 3D Features of Inositol 1,4,5-Trisphosphate Receptor (IP_3_R) Inhibitors in Cancer

**DOI:** 10.3390/ijms222312993

**Published:** 2021-11-30

**Authors:** Humaira Ismatullah, Ishrat Jabeen

**Affiliations:** Research Centre for Modelling and Simulation (RCMS), National University of Sciences and Technology (NUST), Sector H-12, Islamabad 44000, Pakistan; hismatullah.phd15@rcms.nust.edu.pk

**Keywords:** IP_3_R-mediated Ca^2+^ signaling, IP_3_R modulators, pharmacophore modeling, virtual screening, hits, GRIND model, PLS co-efficient correlogram

## Abstract

Inositol 1, 4, 5-trisphosphate receptor (IP_3_R)-mediated Ca^2+^ signaling plays a pivotal role in different cellular processes, including cell proliferation and cell death. Remodeling Ca^2+^ signals by targeting the downstream effectors is considered an important hallmark in cancer progression. Despite recent structural analyses, no binding hypothesis for antagonists within the IP_3_-binding core (IBC) has been proposed yet. Therefore, to elucidate the 3D structural features of IP_3_R modulators, we used combined pharmacoinformatic approaches, including ligand-based pharmacophore models and grid-independent molecular descriptor (GRIND)-based models. Our pharmacophore model illuminates the existence of two hydrogen-bond acceptors (2.62 Å and 4.79 Å) and two hydrogen-bond donors (5.56 Å and 7.68 Å), respectively, from a hydrophobic group within the chemical scaffold, which may enhance the liability (IC_50_) of a compound for IP_3_R inhibition. Moreover, our GRIND model (PLS: Q^2^ = 0.70 and R^2^ = 0.72) further strengthens the identified pharmacophore features of IP_3_R modulators by probing the presence of complementary hydrogen-bond donor and hydrogen-bond acceptor hotspots at a distance of 7.6–8.0 Å and 6.8–7.2 Å, respectively, from a hydrophobic hotspot at the virtual receptor site (VRS). The identified 3D structural features of IP_3_R modulators were used to screen (virtual screening) 735,735 compounds from the ChemBridge database, 265,242 compounds from the National Cancer Institute (NCI) database, and 885 natural compounds from the ZINC database. After the application of filters, four compounds from ChemBridge, one compound from ZINC, and three compounds from NCI were shortlisted as potential hits (antagonists) against IP_3_R. The identified hits could further assist in the design and optimization of lead structures for the targeting and remodeling of Ca^2+^ signals in cancer.

## 1. Introduction

Inositol 1, 4, 5-trisphosphate receptor (IP_3_R)-mediated Ca^2+^ signaling is an important regulatory factor in cancer progression, including invasiveness and cell proliferation [1,2,3]. In carcinogenesis, the Ca^2+^ signals are remodeled to regulate the cell cycle by inducing the early response genes (JUN and FOS) in the G_1_ phase and have a direct influence on cell death [2,3,4]. Thus, the response of malignant cell is overwhelmed by Ca^2+^ signaling by providing them an unconditional advantage of unrestricted cell multiplication and proliferation [5,6], avoiding programmed cell death [7,8], and providing specific adaptations to limited cellular conditions. Therefore, Ca^2+^ signals are known to facilitate metastasis from the primary point of initiation [9,10]. Nevertheless, remodeling of Ca^2+^ signaling by downstream Ca^2+^-dependent effectors is considered a prime reason for sustaining the cancer hallmark [11,12].

Cancer cells rely on the constitutive Ca^2+^ transfer from the endoplasmic reticulum (ER) to mitochondria to sustain their high stipulation of building blocks for ATP production and proteins. Similarly, cancer cells also manipulate the mitochondrial tricarboxylic acid (TCA) cycle and mitochondrial oxidative phosphorylation process to meet their anabolic demands [13,14]. In addition to the pro-invasive and pro-apoptotic role, the overexpression of IP_3_Rs was associated with various cancer types [15]. Among three isoforms of IP_3_R (R_1_, R_2,,_ and R_3_), the subtype IP_3_R_3_ is considered a leading participant in carcinogenesis, since its expression level is associated with the aggressive behavior of colorectal carcinoma cells [16]. Inhibition of IP_3_R_3_ results in a decreased level of cell proliferation in breast cancer [17] and reduced invasion, cell migration, and survival rates in glioblastoma cells [18].

Briefly, the inositol 1,4,5-trisphosphate receptor (IP_3_R), an endoplasmic reticulum (ER) resident intracellular Ca^2+^ release channel, is an essential determinative for Ca^2+^-dependent cellular processes [19,20]. Structurally, each IP_3_R molecule in a tetramer is categorized as a large subunit forming a single channel (Ca^2+^ ion-permeable) with a single IP_3_-binding site [21,22,23,24]. Further, IP_3_ receptor protein can be subdivided into a cytosolic domain and a Ca^2+^ channel domain [25,26]. All of the crucial functional sites responsible for the regulation and function of receptor protein are located in the cytosolic domain. These include an IP_3_-binding core (IBC) region and a suppressor domain (residues ~600) at the N terminus of the protein. The cytosolic domain also includes a central modulatory region (which mostly interacts with regulatory proteins) and a channel (pore) with six putative transmembrane (TM) domains (residues 2276–2589) near the protein’s C terminus [23,27,28,29]. Recent structural investigations of IP_3_Rs [26,30] and availability of the 3D structure of IP_3_R_3_ in apostate and ligand-bound states [30,31] paved the way to study the binding hypothesis of the IP_3_ molecule and antagonists to elucidate the effect of IP_3_R inhibition upon channel gating.

Depending upon the micro-environment of the cell, inhibition of IP_3_R-mediated Ca^2+^ signal activates autophagy as a pro-survival or pro-death response in normal healthy cells [32,33]. Furthermore, pharmacological inhibition of IP_3_R signaling in tumorigenic cells is the key player to impair mitochondrial bioenergetics resulting in the activation of AMP-kinases (AMPK), successively leading towards autophagy followed by necrotic cell death [17,33]. Deficiency in mitochondrial substrates results in the cell death of cancer cells independent of oxidative stress or autophagy as reported by Cárdenas et al. [33].

Considering the importance of IP_3_R-mediated Ca^2+^-signaling inhibition in cancer cells, in the present study, a ligand-based pharmacophore model was generated to identify important features of antagonists that are essential for interaction with the receptor. Further, the virtual screening (VS) was performed based upon the pharmacophore model to identify new potential hits against IP_3_R. The application of GRIND in many computational drug discovery pipelines is evident, including molecular-docking studies [34], 3D-QSAR analysis [35], metabolism profiling [36], molecular kinetics [37,38], ADME prediction, and high-throughput virtual screening [39]. Previously, no predictive QSAR models against IP_3_R antagonists were reported due to the availability of limited and structurally diverse datasets. Therefore, in the present study, alignment-independent molecular descriptors based on molecular interaction fields (MIFs) were used to probe the 3D structural features of IP_3_R antagonists. Additionally, a grid-independent molecular descriptor (GRIND) model was developed to evaluate the proposed pharmacophore model and to establish a binding hypothesis of antagonists with IP_3_R. Overall, this study may add value to recognize the essential pharmacophoric features and their mutual distances and to design new potent ligands required for IP_3_R inhibition.

## 2. Results

### 2.1. Preliminary Data Analysis and Template Selection

**Figure 1:**
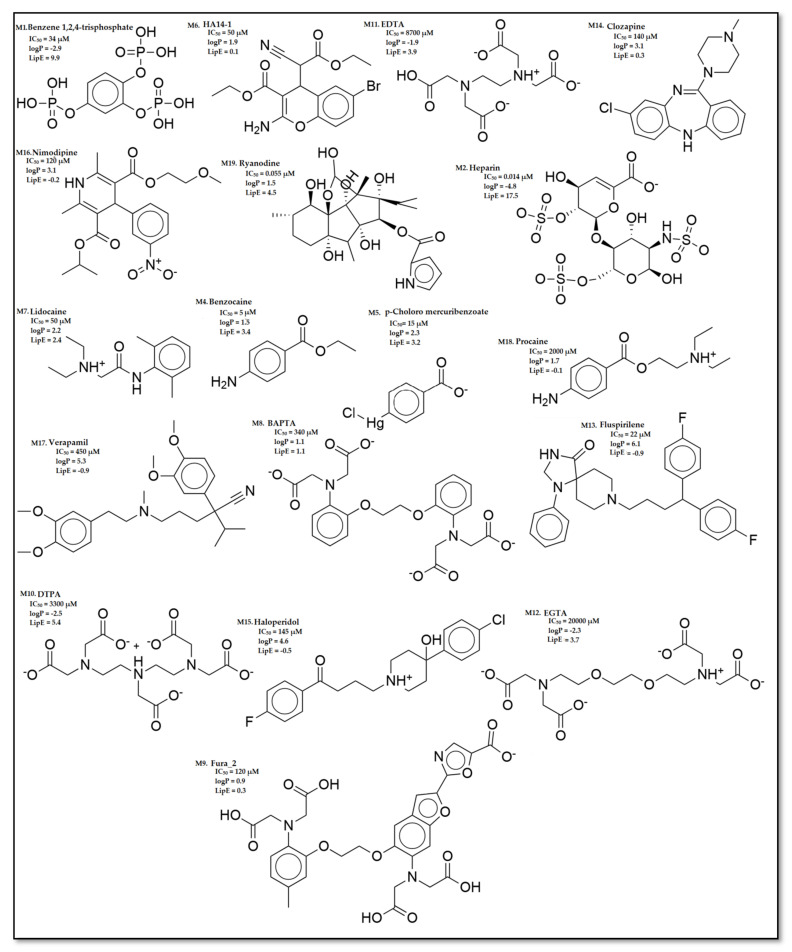
Chemical structure of the compounds in Class M with inhibitory potency (IC_50_) and lipophilic efficiency (LipE) values.

Overall, the dataset of 40 competitive compounds exhibiting 0.0029 µM to 20,000 µM half-maximal inhibitory concentration (IC_50_) against IP_3_R was selected from the ChEMBL database [40] and literature. Based upon a common scaffold, the dataset was divided into four classes (Table 1). Class A consisted of inositol derivatives, where phosphate groups with different stereochemistry are attached at positions R_1–_R_6_. Similarly, Class B consisted of cyclic oxaquinolizidine derivatives commonly known as xestospongins, whereas, Class C was composed of biphenyl derivatives, where phosphate groups are attached at different positions of the biphenyl ring (Table 1). However, Class M consisted of structurally diverse compounds. The chemical structures of Class M are illustrated in Figure 1.

By careful inspection of the activity landscape of the data, the activity threshold was defined as 160 µM (Table S1). The inhibitory potencies (IC_50_) of most actives in the dataset ranged from 0.0029 µM to 160 µM, whereas inhibitory potency (IC_50_) of least actives was in the range of 340 µM to 20,000 µM. The LipE values of the dataset were calculated ranging from −2.4 to 17.2. The physicochemical properties of the dataset are illustrated in Figure S1.

### 2.2. Pharmacophore Model Generation and Validation

**Figure 2:**
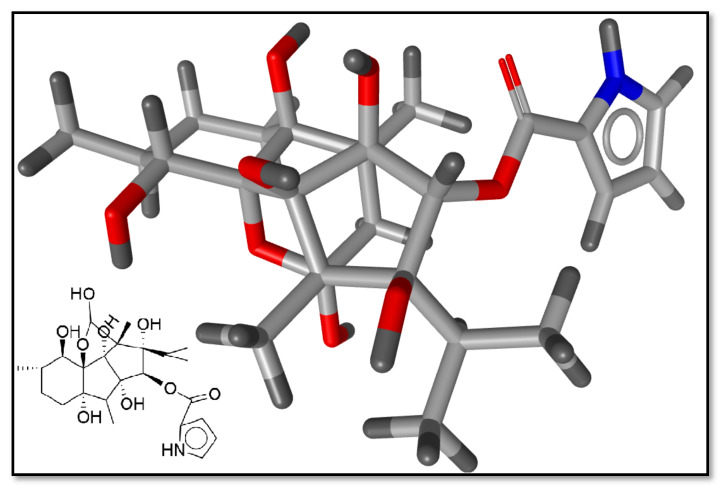
The 3D molecular structure of ryanodine (template) molecule.

Previously, different studies proposed that a range of clogP values between 2.0 and 3.0 in combination with lipophilic efficiency (LipE) values greater than 5.0 are optimal for an average oral drug [48,49,50,51]. By this criterion, ryanodine (IC_50_: 0.055 µM) with a clogP value of 2.71 and LipE value of 4.6 (Table S1) was selected as a template for the pharmacophore modeling (Figure 2). A lipophilic efficacy graph between clogP versus pIC_50_ is provided in Figure S2.

Briefly, to generate ligand-based pharmacophore models, ryanodine was selected as a template molecule. The chemical features within the template, e.g., the charged interactions, lipophilic regions, hydrogen-bond acceptor and donor interactions, and steric exclusions, were detected as important pharmacophoric features. Thus, 10 pharmacophore models were generated by using the radial distribution function (RDF) code algorithm [52]. Once models were generated, each model was validated internally by performing the pairing between pharmacophoric features of the template molecule and the rest of the data to create geometric transformations based upon minimal squared distance deviations [53]. The generated models with the chemical features, the distances within these features, and the statistical parameters to validate each model are shown in Table 2.

Overall, in ligand-based pharmacophore models, hydrophobic features with hydrogen-bond acceptors and hydrogen-bond donors mapped at variable mutual distances (Table 2) were found to be important. Therefore, based on the ligand scout score (0.68) and Matthew’s correlation coefficient (MCC: 0.76), the pharmacophore model 1 was finally selected for further evaluation. The model was generated based on shared-feature mode to select only common features in the template molecule and the rest of the dataset. Based on 3D pharmacophore characteristics and overlapping of chemical features, the model score was calculated. The conformation alignments of all compounds (calculated by clustering algorithm) were clustered based upon combinatorial alignment, and a similarity value (score) was calculated between 0 and 1 [54]. Finally, the selected model (model 1, Table 2) exhibits one hydrophobic, two hydrogen-bond donor, and two hydrogen-bond acceptor features. The true positive rate (TPR) of the final model determined by Equation (4) was 94% (sensitivity = 0.94), and true negative rate (TNR) determined by Equation (5) was 86% (specificity = 0.86). The tolerance of all the features was selected as 1.5, while the radius differed for each feature. The hydrophobic feature was selected with a radius of 0.75, the hydrogen-bond acceptor (HBA_1_) has a 1.0 radius, and HBA_2_ has a radius of 0.5, while both hydrogen-bond donors (HBD) have 0.75 radii. The hydrophobic feature in the template molecule was mapped at the methyl group present at one terminus of the molecule. The carbonyl oxygen present within the scaffold of the template molecule is responsible for hydrogen-bond acceptor features. However, the hydroxyl group may act as a hydrogen-bond donor group. The richest spectra about the chemical features responsible for the activity of ryanodine and other antagonists were provided by model 1 (Figure S3).

The final ligand-based pharmacophore model emphasized that, within a chemical scaffold, two hydrogen-bond acceptors must be separated by a shorter distance (of not less than 2.62 Å) compared to two hydrogen-bond donors (may be 6.97 Å). Additionally, the distance between a hydrogen-bond acceptor and a hydrogen-bond donor should not exceed 3.11–5.58 Å. Moreover, the existence of two hydrogen-bond acceptors (2.62 Å and 4.79 Å) and two hydrogen-bond donors (5.56 Å and 7.68 Å) mapped from a hydrophobic group (yellow circle in Figure S3) within the chemical scaffold may enhance the liability (IC_50_) of a compound for IP_3_R inhibition.

The finally selected pharmacophore model was validated by an internal screening of the dataset and a satisfactory MCC = 0.76 was obtained, indicating the goodness of the model. A receiver operating characteristic (ROC) curve showing specificity and sensitivity of the final model is illustrated in Figure S4. However, for a predictive model, statistical robustness is not sufficient. A pharmacophore model must be predictive to the external dataset as well. The reliable prediction of an external dataset and distinguishing the actives from the inactive are considered critical criteria for pharmacophore model validations [55,56]. An external set of 11 compounds (Figure S5) defined in the literature [57,58,59] to inhibit the IP_3_-induced Ca^2+^ release was considered to validate our pharmacophore model. Our model predicted nine compounds as true positive (TP) out of 11, hence showing the robustness and productiveness (81%) of the pharmacophore model.

### 2.3. Pharmacophore-Based Virtual Screening

**Figure 3:**
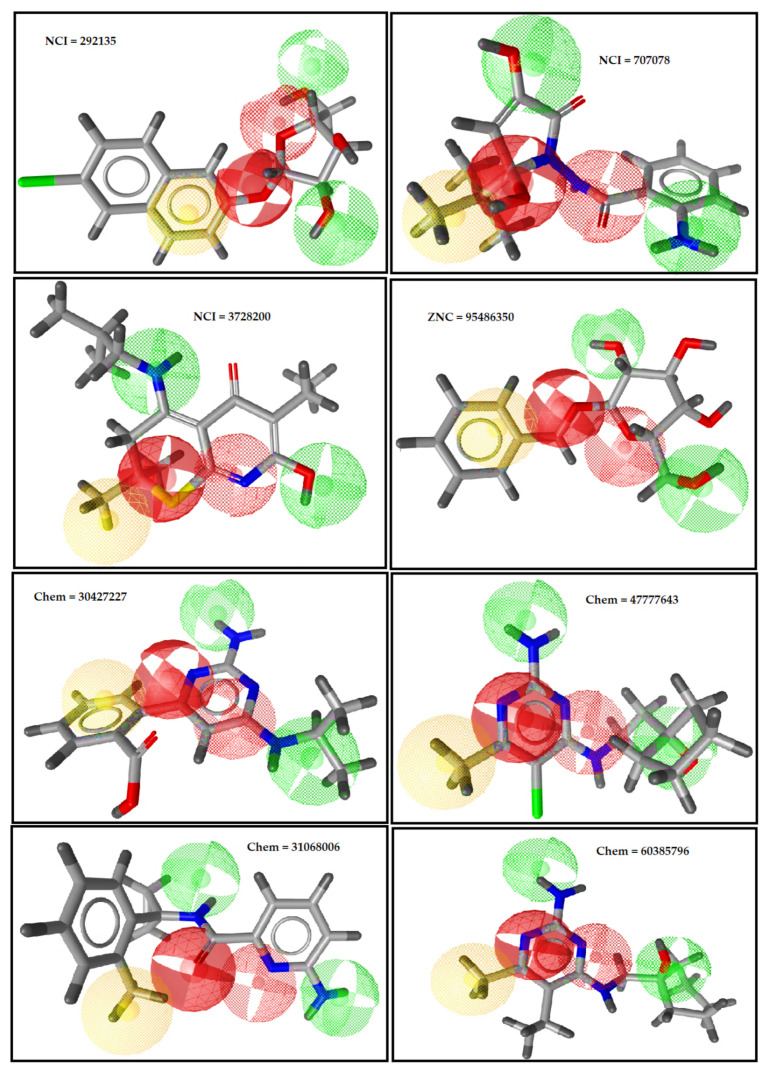
Potential hits (IP_3_R modulators) identified by virtual screening (VS) of National Cancer Institute (NCI) database, ZINC database, and ChemBridge database. After application of several filters and pharmacophore-based virtual screening, these compounds were shortlisted as IP_3_R potential inhibitors (hits). These hits (IP_3_R antagonists) are showing exact feature match with the final pharmacophore model.

In the drug discovery pipeline, virtual screening (VS) is a powerful method to identify new hits from large chemical libraries/databases for further experimental validation. The final ligand-based pharmacophore model (model 1, Table 2) was screened against 735,735 compounds from the ChemBridge database [60], 265,242 compounds in the National Cancer Institute (NCI) database [61,62], and 885 natural compounds from the ZINC database [63]. Initially, the inconsistent data was curated and preprocessed by removing fragments (MW < 200 Da) and duplicates. The biotransformation of the 70–80% drugs was carried out by cytochromes P450 (CYPs), as they are involved in pharmacodynamics variability and pharmacokinetics [63]. The five cytochromes P450 (CYP) isoforms (CYP 1A2, 2C9, 2C19, 2D6, and 3A4) are most important in human drug metabolism [64]. Thus, to obtain non-inhibitors, the CYPs filter was applied by using the Online Chemical Modeling Environment (OCHEM) [65]. The shortlisted CYP non-inhibitors were subjected to a conformational search in MOE 2019.01 [66]. For each compound, 1000 stochastic conformations [67] were generated. To avoid hERG blockage [68,69], these conformations were screened against a hERG filter [70]. Briefly, after pharmacophore screening, four compounds from the ChemBridge database, one compound from the ZINC database, and three compounds from the NCI database were shortlisted (Figure S6) as hits (IP_3_R modulators) based upon an exact feature match (Figure 3). A detailed overview of the virtual screening steps is provided in Figure S7**.**

The current prioritized hits (antagonists) were based upon a data-driven pipeline in the early stages of the drug design process that however, require bioactivity data against IP_3_R.

### 2.4. Molecular-Docking Simulation and PLIF Analysis

**Figure 4:**
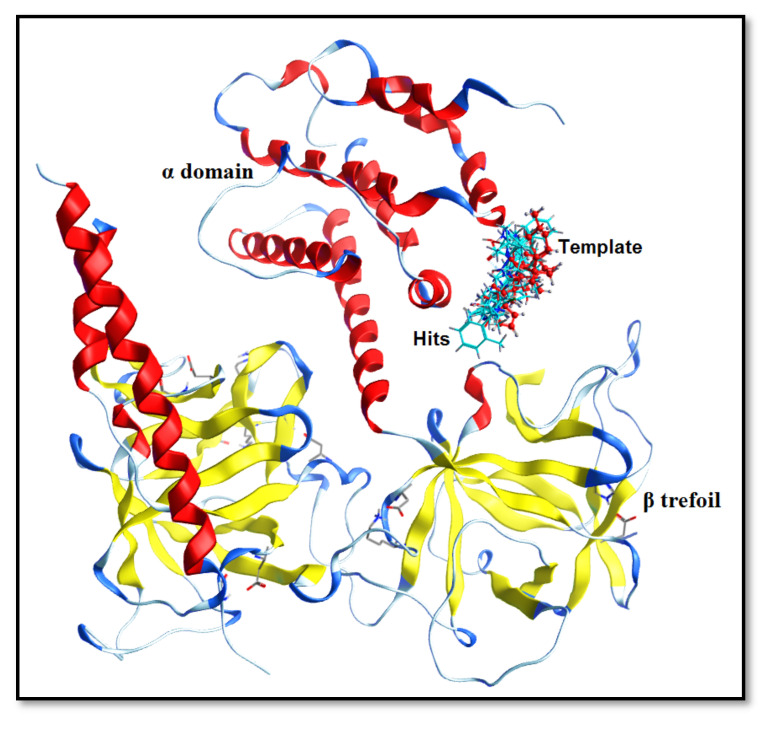
The docking orientation of shortlisted hits in the IP_3_R_3_-binding domain. The secondary structure of the IP_3_R_3_-binding domain is presented where the α domain, β-trefoil region, and turns are presented in red, yellow, and blue, respectively. The template molecule (ryanodine) is shown in red (ball and stick), and the hits are shown in cyan (stick).

Briefly, the top-scored binding poses of each hit (Figure 3) were selected for protein–ligand interaction profile analysis using PyMOL 2.0.2 molecular graphics system [71]. Overall, all the hits were positioned within the α-armadillo domain and β-trefoil region of the IP_3_R_3_-binding domain as shown in Figure 4. The selected hits displayed the same interaction pattern with the conserved residues (arginine and lysine) [19,26,72] as observed for the template molecule (ryanodine) in the binding pocket of IP_3_R.

**Figure 5:**
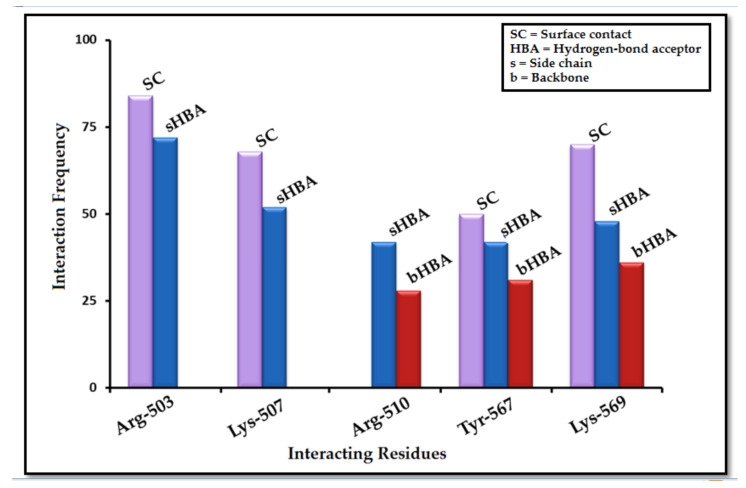
A summarized population histogram based upon occurrence frequency of interaction profiling between hits and the receptor protein. Most of the residues formed surface contact (π–π interactions), whereas some were involved in side chain hydrogen-bond interactions. Overall, Arg-503 and Lys-569 were found to be most interactive residues.

The fingerprint scheme in the protein–ligand interaction profile was analyzed using the Protein–Ligand Interaction Fingerprint (PLIF) tool in MOE 2019.01 [66]. To observe the occurrence frequency of interactions, a population histogram was generated between the receptor protein (IP_3_R_3_) and the shortlisted hit molecules. In the PLIF analysis, the side chain or backbone hydrogen-bond (acceptor or donor) interactions, surface contacts, and ionic interactions were calculated on the basis of distances between atom pairs and their orientation contacts with protein. Our dataset (ligands and hits) revealed the surface contacts (π–π interactions) and hydrogen-bond acceptor and donor (HBA and HBD) interactions with Arg-503, Lys-507, Arg-568, and Lys-569 (Figure S8). Overall, 85% of the docked poses formed either side chain or backbone hydrogen-bond acceptor and donor (HBA and HBD) interactions with Arg-503. Moreover, 73% of the dataset interacted with Lys-569 through surface contacts (π–π interactions) and hydrogen-bond interactions. Similarly, 65% of the hits showed hydrophobic interactions and surface contacts with Lys-507, whereas 50% of the dataset showed π–π interactions and direct hydrogen-bond interactions with Arg-510 and Tyr-567 (Figure 5).

In site-directed mutagenic studies, the arginine and lysine residues were found to be important in the binding of ligands within the IP_3_R domain [72,73], wherein the residues including Arg-266, Lys-507, Arg-510, and Lys-569 were reported to be crucial. The docking poses of the selected hits were further strengthened by previous study where IP_3_R antagonists interacted with Arg-503 (π–π interactions and hydrogen bond), Ser-278 (hydrogen-bond acceptor interactions), and Lys-507 (surface contacts and hydrogen-bond acceptor interactions) [74].

### 2.5. Grid-Independent Molecular Descriptor (GRIND) Analysis

To quantify the relationships between biological activity and chemical structures of the ligand dataset, QSAR is a generally accepted and well-known diagnostic and predictive method. To develop a 3D-QSAR model using GRIND descriptors, three sets of molecular conformations (provided in supporting information in the Materials and Methods section) of the training dataset were subjected independently as input to the Pentacle version 1.07 software package [75], along with their inhibitory potency (pIC_50_) values. To identify more important pharmacophoric features at VRS and to validate the ligand-based pharmacophore model, a partial least square (PLS) model was generated. The partial least square (PLS) method correlated the energy terms with the inhibitory potencies (pIC_50_) of the compounds and found a linear regression between them. The variation in data was calculated by principal component analysis (PCA) and is described in the supporting information in the Results section (Figure S9).

Overall, the energy minimized and standard 3D conformations did not produce good models even after the application of the second cycle of the fractional factorial design (FFD) variable selection algorithm [76]. However, the induced fit docking (IFD) conformational set of data revealed statistically significant parameters. Independently, three GRIND models were built against each previously generated conformation, and the statistical parameters of each developed GRIND model were tabulated (Table 3). 

**Figure 6:**
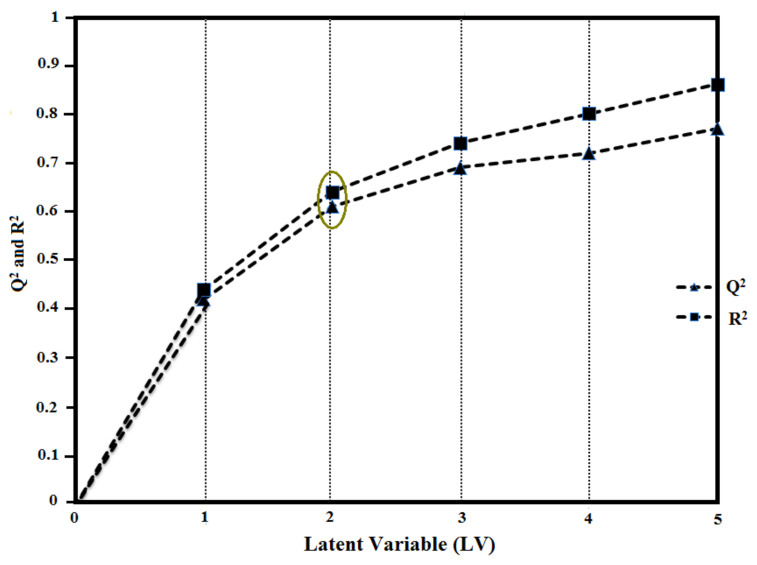
Correlation plot between Q^2^ and R^2^ values of the GRIND model developed by induced fit docking (IFD) conformations at latent variables (LV 1–5). The final GRIND model was selected at latent variable 2.

Therefore, based upon the statistical parameters, the GRIND model developed by the induced fit docking conformation was selected as the final model. Further, to eliminate the inconsistent variables from the final GRIND model, a fractional factorial design (FFD) variable selection algorithm [76] was applied, and statistical parameters of the model improved after the second FFD cycle with Q^2^ of 0.70, R^2^ of 0.72, and standard deviation of error prediction (SDEP) of 0.9 (Table 3). A correlation graph between the latent variables (up to the fifth variable, LV_5_) of the final GRIND model versus Q^2^ and R^2^ values is shown in Figure 6. The R^2^ values increased with the increase in the number of latent variables and a vice versa trend was observed for Q^2^ values after the second LV. Therefore, the final model at the second latent variable (LV_2_), showing statistical values of Q^2^ = 0.70, R^2^ = 0.72, and standard error of prediction (SDEP) = 0.9, was selected for building the partial least square (PLS) model of the dataset to probe the correlation of structural variance in the dataset with biological activity (pIC_50_) values.

Briefly, partial least square (PLS) analysis [77] was performed by using leave-one-out (LOO) as a cross-validation procedure [78] to correlate the 3D molecular structure features with the inhibitory potency (pIC_50_) values against IP_3_R. Furthermore, a plot of actual versus predicted inhibitory potency (pIC_50_) values obtained after multiple linear regression analysis using the leave-one-out (LOO) cross-validation [78,79] of the training dataset is illustrated in Figure S10 in the Results section. The model was validated by using cross-validation methods [79], including the leave-five-out (LFO) method (Table S2). The actual and predicted inhibitory potency values (pIC_50_) of the training and test datasets with the residual differences were also tabulated (Tables S3 and S4). All the compounds in the training set (R^2^ = 0.76), as well as in the test set (R^2^ = 0.65), were predicted with a residual difference of ±2 log units.

**Figure 7:**
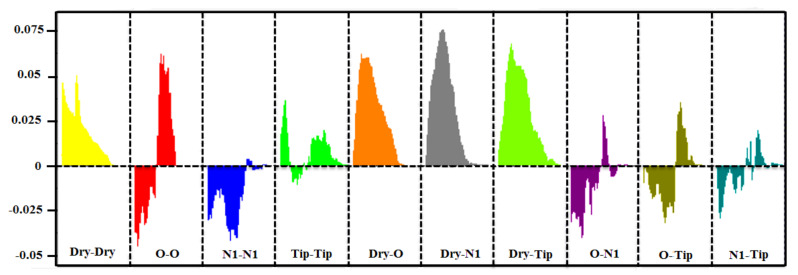
Partial least square (PLS) coefficient correlogram plot representing direct (positive values) and inverse (negative values) correlations of the GRIND variables with inhibitory potency (pIC_50_) against IP_3_R antagonists.

Moreover, the partial least square (PLS) coefficients correlogram (Figure 7) containing auto (Dry-Dry, Tip-Tip, O-O, and N1-N1) and cross variables (Dry-O, Dry-Tip, Dry-N1, Tip-O, Tip-N1, O-N1) correlated positively and negatively with the inhibitory potency (pIC_50_) of IP_3_R. Noticeably, Dry-Dry, Dry-O, Dry-N1, and Dry-Tip variables correlated positively and had a major influence in defining the inhibitory potency of a compound against IP_3_R. However, the N1-N1 variable corresponded negatively to the biological activity (pIC_50_) and depicted the more prominent 3D structural feature in the least potent inhibitors of the dataset.

**Figure 8:**
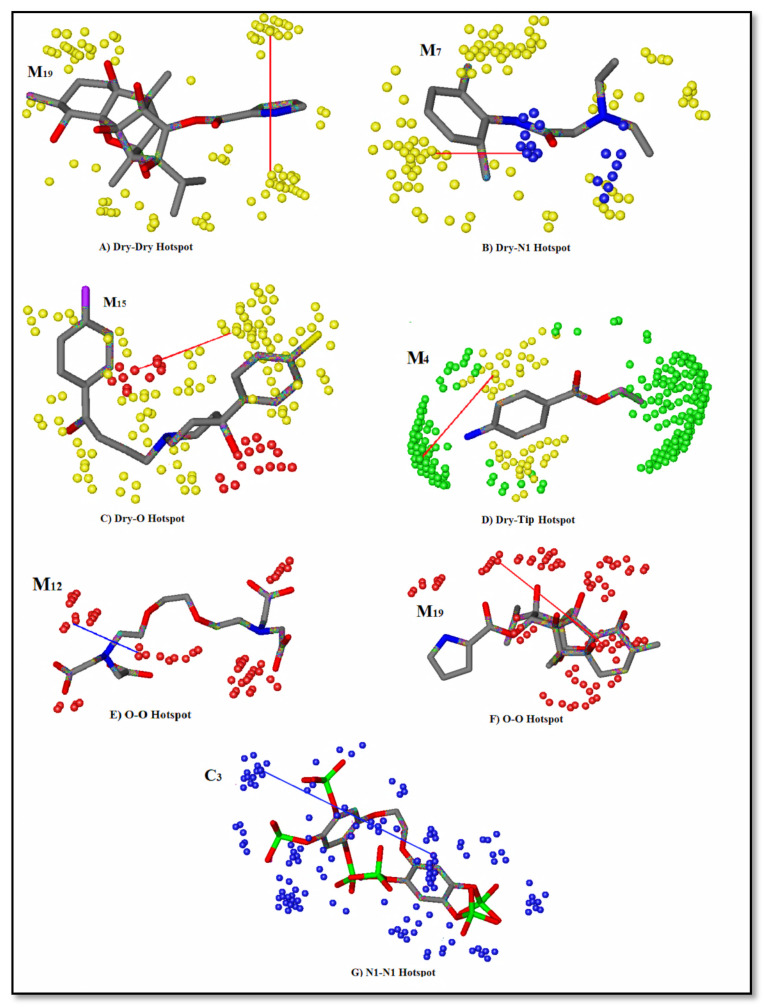
(**A**) Dry-Dry probes represent the presence of hydrophobic moiety within the highly active compounds (0.0029–160 µM) at a distance of 6.4–6.8 Å, and (**B**) represents the Dry-N1 set of probes within a hydrophobic region and a hydrogen-bond acceptor group (nitrogen of M_7_) present at a mutual distance of 7.6–8.0 Å in highly active compounds. Similarly, (**C**) reflects the presence of a hydrophobic region and a hydrogen-bond donor (oxygen of M_15_) contour designated by a Dry-O peak in the correlogram at a mutual distance of 6.8–7.2 Å. (**D**) depicts the Dry-Tip pair of probes describing the presence of a hydrophobic contour in combination with a steric hotspot separated by a mutual distance of 5.60–6.00 Å in highly active compounds. (**E**) represents the O-O probes defining the two hydrogen-bond donor groups at a shorter distance of 2.4–2.8 Å present in the least active compounds and implicating a negative effect on the inhibitory potency of a compound against IP_3_R, and (**F**) shows the positive effect of two hydrogen-bond donor contours (O-O probe) separated by a larger distance ranging from 10.4–10.8 Å in the molecule (M_19_). This was present in all active compounds (0.0029–160 µM) of the dataset. (**G**) represents the N1-N1 probe indicating the presence of two hydrogen-bond acceptor hotspots in a molecule at a mutual distance of 9.2–9.8 Å, surrounding the data with the least inhibition potential (IC_50_) values between 2000 and 20,000 µM.

**Figure 9:**
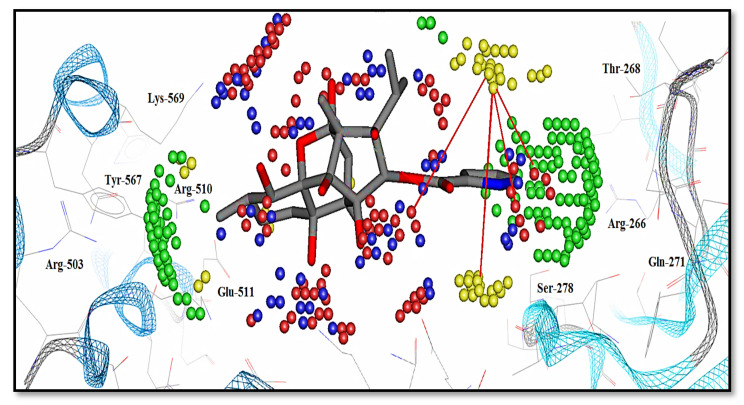
Representing the important hotspots (contours define the virtual receptor site (VRS)) identified by the GRIND model for the high inhibitory potency of antagonist–IP_3_R interaction. Yellow contour defines the hydrophobic region present in the binding pocket. The presence of a ring structure against Arg-266 and Arg-270 complemented the hydrophobic (π–π) interactions. Similarly, blue contour defines the hydrogen-bond acceptor group complementing the presence of side chains of Arg-510 and Tyr-567 residues. The amide group of Arg-510 in the binding pocket of IP_3_R complemented the hydrogen-bond acceptors contour.

More explicitly, the Dry-Dry auto variable (Figure 7) represented the pair of two hydrophobic nodes interacting favorably at a mutual distance of 6.4–6.8 Å at the virtual receptor site (VRS). Since the present data was a set of diverse compounds, many such variables were found in all active compounds (0.0029–160 µM) within a defined distance. Additionally, at a shorter distance of 5.20–5.60 Å, this variable was present in the moderately active compound M_9_ (120 µM). Mostly, the active compounds consisted of two or more aromatic rings. However, more than two rings (aromatic moieties or aryl) were present in the M_19_ structure (Figure 8A) and created a hydrophobic cloud surrounding the ring and provided a significant basis for the hydrophobic (π–π/surface contact) interactions. Further, the presence of nitrogen at the ortho position of the ring may facilitate the aromatic feature (Dry) at the virtual receptor site (VRS). Similarly, the Arg-266, Ser-278, Arg-510, and Tyr-567 residues present in the binding core of IP_3_R were found to be involved in the hydrophobic interactions (Figure 9). Previously, Arg-266 was determined as an important facilitator of hydrophobic interactions [74].

Similarly, the Dry-N1 probe in the correlogram (Figure 7) was positively correlated with the activity of the compound against IP_3_R. It depicted a hydrophobic and a hydrogen-bond donor hotspot at a distance of 7.6–8.0 Å in the virtual receptor site (VRS). Most of the active compounds, M_19_, M_4,_ and M_7_ (0.0029–160 µM), in the dataset were characterized by having carbonyl oxygen attached with ring structures (Figure 8B). The presence of a hydrogen-bond acceptor group at a distance of 4.79 Å from the hydrophobic feature of the template molecule was identified as an important feature in defining the inhibitory potency of a compound by our ligand-based pharmacophore model (Table 4). The difference in distances can be correlated to the mapped virtual site receptor in the GRIND versus ligand features in the pharmacophore modeling. Furthermore, the IP_3_R-binding core (IBC) had a predominantly positive electrostatic potential where hydrogen-bond (acceptor and donor) and ionic interactions were facilitated by multiple basic amino acid residues [44]. The Glu-511 residue may provide a proton from its carboxyl group in the receptor-binding site and complemented the hydrogen-bond donor contour predicted by GRIND (Figure 9). Similarly, the Lys-569 residue and the α-amino nitrogen group found in the side chains of Arg-510, Arg-266, and Arg-270 harbored the ryanodine ligand by enabling the hydrogen-bond donor and acceptor interactions.

Further, the Dry-O peak in the correlogram (Figure 7) represented the hydrogen-bond acceptor contour at a distance of 6.8–7.2 Å from the hydrophobic region in the VRS. The M_19_ and M_15_, the most active compounds (0.0029–160 µM) of the dataset, consisted of protonated nitrogen in the ligand structure (Figure 8C) that provided hydrogen-bond donor characteristics complementing the hydrogen-bond acceptor contour at the virtual receptor site. Also, the hydroxyl group found on the side chain of the template molecule may exhibit hydrogen-bond donor qualities. Furthermore, in the ligand-based pharmacophore model, the hydrogen-bond donor (HBD) group present at a distance of 5.56 Å from the hydrophobic feature seemed to be a more influential one in defining the inhibitory potency of IP_3_R (Table 4). This further strengthened the authenticity of our GRIND model outcomes. The presence of a hydrogen-bond acceptor complemented the α-amino nitrogen group found in the side chain of Arg-510 and the polar amino acid residue Tyr-567 in the binding core of IP_3_R. However, Tyr-567 facilitated the hydrogen-bond donor and acceptor interactions simultaneously. In the receptor-binding site, the side chains of Ser-278, Lys-507, and Lys-569 complemented the presence of hydrogen-bond acceptor contours predicted by GRIND in the virtual receptor site (Figure 9).

Furthermore, the presence of a hydrophobic moiety and a steric hotspot at a mutual distance of 5.60–6.00 Å in VRS defining the 3D molecular shape of the antagonists is represented by the Dry-Tip peak in the correlogram (Figure 7). The ring (aryl/aromatic) structure present in most of the compounds represented the hydrophobic characteristics of the particular compound (Figure 8D). Here, the molecular boundaries of the hydrophobic groups were suggested with the combination of a steric hotspot. Considering the important role of Arg-266 and Arg-510 in the binding core of IP_3_R [74], the presence of a steric hotspot along with a hydrophobic region represented the hydrophobic interactive nature of the receptor-binding site. The shape complementarity of the Tip contour defined by GRIND may be supported by the presence of Arg-266 in the β-trefoil (226–435) region and Tyr-567 in the α-helix (436–604) region of the IP_3_R-binding core (Figure 9) [30,31]. The two structurally distinct domains, β-trefoil and α-armadillo repeats, created an L-shaped cleft structure generated by the perpendicular position of the two domains and surrounded by a cluster of several basic amino acids, forming the InsP_3_-binding site [26]. Interestingly, the curved molecular boundary at a longer distance of 16.40 Å–16.80 Å exhibited a significant impact in defining a compound’s inhibitory potency as compared to the linear-shaped boundary at a shorter distance of 10.00 Å–10.40 Å (Figure S11). Overall, the hydrophobic region (Dry in GRIND and Hyd in ligand-based pharmacophore) seemed to be the most important contour, as the other pharmacophoric features (including a hydrogen-bond donor (N1), a hydrogen-bond acceptor (O) contour, and the steric molecular hotspot (Tip)), were mapped and all distances were calculated from this region.

Moreover, the correlogram (Figure 7) indicated the O-O auto probe, at a shorter distance of 2.4–2.8 Å, was negatively correlated (Figure 8E), while at a longer distance of 10.4–10.8 Å, it was positively correlated (Figure 8F) with the inhibitory potency of a compound against IP_3_R. In the present dataset, the presence of the nitrogen and hydroxyl groups complemented the presence of two hydrogen-bond donor contours in compounds having IC_50_ in the range of 93 µM to 160 µM (moderately active). In the receptor-binding site, the presence of two hydrogen-bond acceptors at a wider range was augmented by the presence of side chains of Ser-278, Lys-507, and Lys-569 (Figure 9). Our ligand-based pharmacophore model also substantiated the existence of two hydrogen-bond donor groups at a distance of 6.97 Å that played an important role in defining the inhibitory potency of a molecule against IP_3_R.

In the partial least square (PLS) correlogram (Figure 7), the N1-N1 contour was negatively correlated with the activity of compounds, defining the presence of two hydrogen-bond donor contours at a mutual distance of 9.2–9.8 Å in VRS. The compounds with the least inhibition potential (IC_50_) values between 2000 and 20,000 µM had diverse scaffold structures and one to four hydrogen-bond acceptor groups complementing the N1-N1 hotspot region (Figure 8G). However, none of the active compounds (0.0029–160 µM) in the dataset showed the N1-N1 hotspot, mainly due to the absence of a second hydrogen-bond acceptor group. Thus, the presence of two hydrogen-bond acceptor groups complementing the N1-N1 (hydrogen-bond donor) probe at a distance of 9.2–9.8 Å may reduce the IP_3_R inhibition potential.

Taking into account the combined pharmacophore model and the GRIND, the presence of a hydrogen-bond acceptor (4.79 Å) and a hydrogen-bond donor (5.56 Å) group mapped from a hydrophobic feature within the chemical scaffold of a compound may be responsible for enhanced inhibitory potency against IP_3_R. Similarly, the presence of a hydrogen-bond donor and hydrogen-bond acceptor groups at a distance of 7.6–8 Å and 6.8–7.2 Å, respectively, mapped from a hydrophobic hotspot having a particular hydrophobic edge (Tip) in the virtual receptor site may be associated with the increase of the biological activity of IP_3_R inhibitors. In the receptor-binding site, the α-amino nitrogen group found in the side chain of Arg-510 and the polar amino acid residue Tyr-567 in the binding pocket of IP_3_R facilitated the hydrogen-bond acceptor interactions (Figure 9). Furthermore, Tyr-567 residue showed the hydrogen-bond donor and acceptor interactions simultaneously, whereas Glu-511 may provide a proton from its carboxyl group in the receptor-binding site and complement the hydrogen-bond donor contours. Moreover, Arg-266, Tyr-567, and Ser-278 provided the hydrophobic interactions in the binding cavity of IP_3_R. The Tip formed around the ring structure defined the hydrophobic nature of the molecular boundary, as well as the receptor-binding site (Figure 9).

### 2.6. Validation of GRIND Model

The validation of the GRIND model was the most crucial step [80], including the assessment of data quality and the mechanistic interpretability of model applicability, in addition to statistical parameters [81,82]. The performance of the model can be checked by various methods. Conventionally, the GRIND model was assessed by multiple linear regression analysis R^2^ or Ra^2^ (the explained variance) with a threshold value greater than 0.5. However, statistical parameters of models are not always sufficient and acceptable to analyze the model quality and predictive ability. Therefore, further validation techniques are required to validate the developed model quality and optimal predictive ability. The predictive potential of a model can be judged by both internal and external validation methods. For internal validation, conventional methods include the calculation of correlation coefficient (Q^2^), and for external validation, a predictive correlation coefficient (R^2^_-pred_) bearing a threshold of 0.5 [80].

The cross-validation (CV) method is considered a superior method [64,83] over external validation [84,85]. Therefore in this study, the reliability of the proposed GRIND model was validated via cross-validation methods. The leave-one-out (LOO) method of CV yielded a Q^2^ value of 0.61. However, after successive applications of FFD, the second cycle improved the model quality to 0.70. Similarly, the leave-many-out (LMO) method is a more correct one compared to the leave-one-out (LOO) method in CV, specifically when the training dataset is considerably small (≤ 20 ligands) and the test dataset is not available for external validation. The application of the LMO method on our QSAR model produced statistically good enough results (Table S2), although internal and external validation results (if they exhibited a good correlation between observed and predicted data) are considered satisfactory enough. However, Roy and coworkers [81,82,83] introduced an alternative measure r_m_^2^ (modified R^2^) for the selection of the best predictive model. The r_m_^2^ (Equation (1)) is applied to the test set and is based upon the observed and predicted values to indicate the better external predictability of the proposed model.

(1) $$r_{m}{}^{2}=r^{2}\left(1-\left|\sqrt{r^{2}-r_{0}{}^{2}}\;\right|\right)\;\;$$

where **r^2^** shows the correlation coefficient of observed values and **r_0_^2^** is the correlation coefficient of predicted values with the zero intersection axes. The **r_m_^2^** values of the test set were tabulated (Table S4). Good external predictability is considered for the values greater than 0.5 [83].

Moreover, the reliability of the proposed model was analyzed via applicability domain (AD) analysis by using the “applicability domain using standardization approach” application developed by Roy and coworkers [84]. The response of a model (test set) was defined by the characterization of the chemical structure space of the molecules present in the training set. The estimation of uncertainty in predicting a molecule’s similarity (how similar it is with the prediction) to construct a GRIND model is a critical step in the domain of applicability analysis. The GRIND model is only acceptable when the prediction of the model response falls within the AD range. Ideally, a normal distribution [85] pattern must be followed by the descriptors of all compounds in the training set. Thus, according to this rule (distribution), most of the population (99.7%) in the training and test data may exhibit ≤±3 mean of standard deviation (SD) range in the AD. Any compound outside the AD is considered an outlier. In our GRIND model, the SD mean was in the range of ±1, while none of the compounds in the training set or test set was predicted as an outlier (Tables S3 and S4). A detailed computation of the AD analysis is provided in the supplementary file.

## 3. Discussion

Considering the indispensable role of Ca^2+^ signaling in cancer progression, different studies identified the subtype-specific expression of IP_3_R remodeling in many cancers. The significant remodeling and altered expression of IP_3_R were associated with a particular cancer type in many cases [1,86]. However, in some cancer cell lines, the sensitivity of cancer cells toward the disruption of Ca^2+^ signaling was evident, in such a way that, inhibition of IP_3_R-mediated Ca^2+^ signaling may induce cell death instead of pro-survival autophagy response [33,87]. Thus, the inhibition of IP_3_R-mediated Ca^2+^ signaling may represent one of the promising cancer therapies. Even though IP_3_R channels were implicated in a variety of human disorders, the structural basis for signal recognition and gating mechanism is not well known. Despite the recent availability of structural details of IP_3_R [19,31,88], the exact binding mechanism of antagonists within the IP_3_-binding core remains elusive. Therefore, in this study, we hypothesized 3D-binding features of IP_3_R modulators by using combined pharmacoinformatic approaches, including ligand-based pharmacophore modeling, virtual screening, and grid-independent molecular descriptor (GRIND) models.

Our ligand-based pharmacophore model’s results emphasized the presence of a hydrogen-bond acceptor separated from a hydrogen-bond donor group by a distance of 3.64 Å, facilitating the compound to interact more effectively against IP_3_R. Shorter distances between both the hydrogen-bond features (hydrogen-bond acceptor and donor) may result in more binding potential compared to the longer distance. This was further strengthened by our GRIND model, where a longer distance between the hydrogen-bond donor and acceptor group at the virtual receptor site negatively correlated with the inhibiting potency of IP_3_R. Our findings were in consistent with the previously proposed phosphorus–phosphorus distances (4.3 Å), where phosphate groups (interacting as hydrogen-bond acceptors and donors) at positions R_4_ and R_5_ of an AdA (adenophostin A) molecule bound with the PH domain [89]. Our predicted distance varied slightly with the Bosanac et al. findings for the similar pair of phosphate groups, i.e., 5.0 Å. Previously, this distance was revealed to be significant in defining the binding potential of the modulators with IP_3_R [90].

It was also hypothesized from our results that the hydrogen-bond acceptor group and a hydrogen-bond donor group mapped from a hydrophobic feature may enhance the inhibitory potency of a compound against IP_3_R. The presence of a hydrophobic feature within the chemical scaffold and at the virtual receptor site implicated its influential role in determining the inhibition potential of the compound. Thus, it was tempting to conclude that the most important feature in defining the inhibitory potency of a compound against IP_3_R is the hydrophobic feature, as all other features were mapped from this particular feature. Our GRIND model results further reinforced the importance of a hydrophobic feature in the binding core of IP_3_R. Previously, in the α-domain of IP_3_R _(mouse)_, two highly conserved but relatively large surface areas were identified. These conserved areas encompassed a relatively high proportion of aromatic residues that might serve as a hydrophobic interactive site of the receptor [73,90,91]. Moreover, structure-based and site-directed mutagenesis studies demonstrated a key role of arginine and lysine residues in IP_3_R’s binding core, where the Arg-266, Lys-508, and Arg-510 were considerably more crucial in binding [72,92]. Furthermore, it was proposed that the ‘adenophostin A’ modulator interacted within the binding core of IP_3_R more effectively via hydrophobic interactions [89,93,94]. Recently, hydrophobic and surface contacts of antagonists were found with the Arg-266, Thr-268, Ser-278, Lys-507, and Tyr-569 backbone and side-chain amino acid residues. However, Arg-266, Arg-510, and Ser-278 residues were found to be involved in π–π interactions specifically [74].

Similarly, the hydrogen-bond acceptor group (HBA) present at a shorter distance from a hydrophobic feature in the chemical scaffold may exhibit more potential for binding activity compared to the one present at a wider distance. This was further confirmed by our GRIND model by complementing the presence of a hydrogen-bond donor contour (N1) at a distance of 7.6–8 Å from the hydrophobic contour. In the receptor-binding site, this was compatible with the previous studies, where a conserved surface area with mostly positive charged amino acids was found to play an important role in facilitating hydrogen-bond interactions [90,95]. Also, the positive allosteric potential of the IP_3_R-binding core may be due to the presence of multiple basic amino acid residues that facilitated the ionic and hydrogen-bond (acceptor and donor) interactions [88]. Arginine residues (Arg-510, Arg-266, and Arg-270) were predominantly present and broadly distributed throughout the IP_3_R-binding core (Figure S12), providing α-amino nitrogen on their side chains and allowing the ligand to interact via hydrogen-bond donor and acceptor interactions. This was further strengthened by the binding pattern of IP_3_ where residues in β domain-mediated hydrogen-bond interactions by anchoring the phosphate group at position R_4_ within the binding core of IP_3_R [74,90,96]. In previous studies, an extensive hydrogen-bond network was observed between the phosphate group at position R_5_ and Arg-266, Thr-267, Gly-268, Arg-269, Arg-504, Lys-508, and Tyr-569 [74,96,97]. Furthermore, two hydrogen-bond donor groups at a longer distance were correlated with the increased inhibitory potency (IC_50_) of antagonists against IP_3_R. Our GRIND model’s outcomes agreed with the presence of two hydrogen-bond acceptor contours at the virtual receptor site. In the receptor-binding site, the presence of Thr-268, Ser-278, Glu-511, and Tyr-567 residues complemented the hydrogen-bond acceptor properties (Figure S12).

In the GRIND model, the molecular descriptors were calculated in an alignment-free manner, but they were 3D conformational dependent [98]. Docking methods are widely accepted and less demanding computationally to screen large hypothetical chemical libraries to identify new chemotypes that potentially bind to the active site of the receptor. During binding-pose generation, different conformations and orientations of each ligand were generated by the application of a search algorithm. Subsequently, the free energy of each binding pose was estimated using an appropriate scoring function. However, a conformation with RMSD < 2 Å may be generated for some proteins, but this may be less than 40% of conformational search processes. Therefore, the bioactive poses were not ranked up during the conformational search process [99]. In our dataset, a correlation between the experimental inhibitory potency (IC_50_) and binding affinities was found to be 0.63 (Figure S14).

For the confident predictions and acceptability of QSAR models, one of the most decisive steps is the use of validation strategies [100]. The Q^2^_LOO_ with a value slightly higher than 0.5 is not considered a good indicative model, but a highly robust and predictive model is considered to have values not less than 0.65 [83,86,87]. Similarly, the leave-many-out (LMO) method is a more correct one compared to the leave-one-out (LOO) method in cross validation (CV), specifically when the training dataset is considerably small (≤20 ligands) and the test dataset is not available for external validation. Application of the leave-Five-out (LFO) method on our QSAR model produced statistically well enough results (Table S2). For a good predictive model, the difference between R^2^ and Q^2^ must not exceed 0.3. For an indicative and highly robust model, the values of Q^2^_LOO_ and Q^2^_LMO_ should be as similar or close to each other as possible and must not be distant from the fitting value R^2^ [88]. In our validation methods, this difference was less than 0.3 (LOO = 0.2 and LFO = 0.11). Additionally, the reliability and predictive ability of our GRIND model was validated by applicability domain analysis, where none of the compound was identified as an outlier. Hence, based upon the cross-validation criteria and AD analysis, it was tempting to conclude that our model was robust. However, the presence of a limited number of molecules in the training dataset and the unavailability of an external test set limited the indicative quality and predictability of the model.

Thus, based upon our study, we can conclude that a novel or highly potent antagonist against IP_3_R must have a hydrophobic moiety (may be aromatic, benzene ring, aryl group) at one end. There should be two hydrogen-bond donors and a hydrogen-bond acceptor group within the chemical scaffold, distributed in such a way that the distance between the hydrogen-bond acceptor and the donor group is shorter compared to the distance between the two hydrogen-bond donor groups. Moreover, to obtain the maximum potential of the compound, the hydrogen-bond acceptor may be separated from a hydrophobic moiety at a shorter distance compared to the hydrogen-bond donor group.

## 4. Materials and Methods

**Figure 10:**
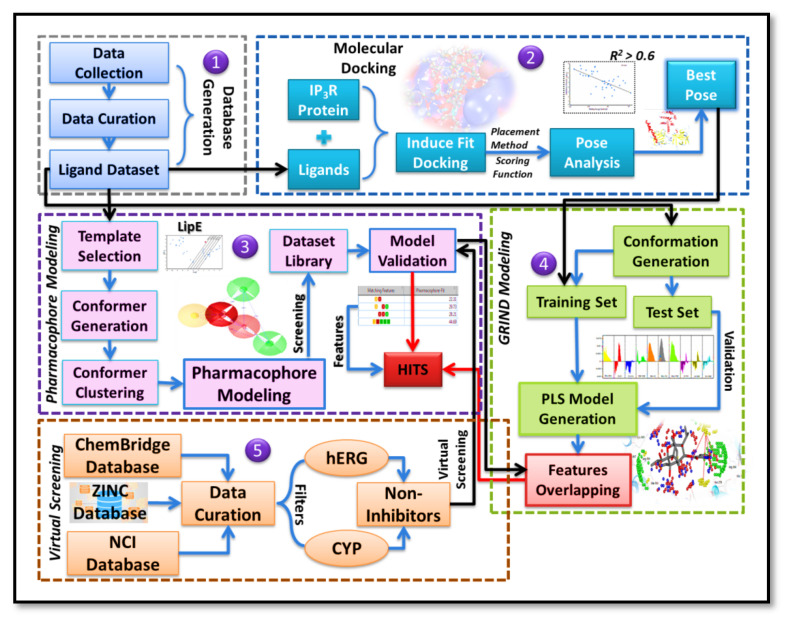
Detailed workflow of the computational methodology adopted to probe the 3D features of IP_3_R antagonists. The dataset of 40 ligands was selected to generate a database. A molecular docking study was performed, and the top-docked poses having the best correlation (R^2^ > 0.5) between binding energy and pIC_50_ were selected for pharmacophore modeling. Based upon pharmacophore model, the ChemBridge database, National Cancer Institute (NCI) database, and ZINC database were screened (virtual screening) by applying different filters (CYP and hERG, etc.) to shortlist potential hits. Furthermore, a partial least square (PLS) model was generated based upon the best-docked poses, and the model was validated by a test set. Then pharmacophoric features were mapped at the virtual receptor site (VRS) of IP_3_R by using a GRIND model to extract common features essential for IP_3_R inhibition.

A detailed overview of methodology has been illustrated in Figure 10.

### 4.1. Ligand Dataset (Collection and Refinement)

A dataset of 23 known inhibitors competitive to the IP_3_-binding site of IP_3_R was collected from the ChEMBL database [40]. Additionally, a dataset of 48 inhibitors of IP_3_R, along with biological activity values, was collected from different publication sources [45,46,101,102,103,104,105]. Initially, duplicates were removed, followed by the removal of non-competitive ligands. To avoid any bias in the data, only those ligands having IC_50_ values calculated by fluorescence assay [106,107] were shortlisted. Figure S13 represents the different data preprocessing steps. Overall, the selected dataset comprised 40 ligands. The 3D structures of shortlisted ligands were constructed in MOE 2019.01 [66]. Furthermore, the stereochemistry of each stereoisomer was corrected and redrawn manually using MarvinSketch 18.8 [108]. The protonation (with 80% solvent) was performed in MOE at pH 7.4, followed by an energy minimization process using the MMFF94x force field [109]. Further, to build a GRIND model, the dataset was divided into a training set (80%) and test set (20%) using a diverse subset selection method as described by Gillet et al. [110] and in various other studies [111,112,113,114,115]. Briefly, 379 molecular descriptors (2D) available in MOE 2019.01 [66] were computed to calculate the molecular diversity of the dataset. To construct the GRIND model, a training set of 33 compounds (80%) was selected while the remaining compounds (20% data) were used as the test set to validate the GRIND model.

### 4.2. Molecular-Docking Simulations

The receptor protein, IP_3_R_3(human)_ (PDB ID: 6DQJ) was prepared by protonating at pH 7.4 with 80% solvent at 310 K temperature in the Molecular Operating Environment (MOE) version 2019.01 [66]. The [6DQJ] receptor protein is a ligand-free protein in a pre-activated state that requires IP_3_ ligand or Ca^+2^ for activation. This ready-to-bound structure was considered for molecular-docking simulations. The energy minimization process with the ‘cut of value’ of 8 was performed by using the AMBER10:EHT force field [116,117]. In molecular-docking simulations, the 40 compounds of the final selected dataset were considered as a ligand dataset, and induced fit docking protocol [118] was used to dock them within the binding pocket of IP_3_R_3_. Previously, the binding coordinates of IP_3_R were defined via mutagenesis studies [72,119]. The amino acid residues in the active site of the IP_3_R_3_ included Arg-266, Thr-267, Thr-268, Leu-269, and Arg-270 positioned at the α domain and Arg-503, Glu-504, Arg-505, Leu-508, Arg-510, Glu-511, Tyr-567, and Lys-569 from the β-trefoil domain. 

Briefly, for each ligand, 100 binding solutions were generated using the default placement method Alpha Triangle and scoring function Alpha HB. To remove bias, the ligand dataset was redocked by using different placement methods and combinations of different scoring functions, such as London dG, Affinity dG, and Alpha HB provided in the Molecular Operating Environment (MOE) version 2019.01 [66]. Based on different scoring functions, the binding energies of the top 10 poses of each ligand were analyzed. The best scores provided by the Alpha HB scoring function were considered (Table S5, docking protocol optimization is provided in supplementary Excel file). Further, the top-scored binding pose of each ligand was correlated with the biological activity (**pIC_50_**) value (Figure S14). The top-scored ligand poses that best correlated (R^2^ > 0.5) with their biological activity (**pIC_50_**) were selected for further analysis.

### 4.3. Template Selection Criteria for Pharmacophore Modeling

Lipophilicity contributes to membrane permeability and the overall solubility of a drug molecule [120]. A calculated log P (**clogP**) descriptor provided by Bio-Loom software [121] was used for the estimation of molecular lipophilicity of each compound in the dataset (Table 1, Figure 1). Generally, in the lead optimization process, increasing lipophilicity may lead to an increase in in vitro biological activity but poor absorption and low solubility in vivo [122]. Therein, normalization of the compound’s activity concerning lipophilicity was considered an important parameter to estimate the overall molecular lipophilic efficiency (**LipE**) (Equation (2)) [123,124].

(2) $$LipE=pIC_{50}-clogP$$

Therefore, the **LipE** values of the present dataset were calculated using a Microsoft Excel spreadsheet as described by Jabeen et al. [50]. From the dataset, a template molecule based upon the active analog approach [55] was selected for pharmacophore model generation. Moreover, to evaluate drug-likeness, the activity/lipophilicity (**LipE**) parameter ratio [125] was used to select the highly potent and efficient template molecule. Previously, different studies proposed an optimal range of **clogP** values between 2 and 3 in combination with a **LipE** value greater than 5 for an average oral drug [48,49,51]. By this criterion, the most potent compound having the highest inhibitory potency in the dataset with optimal **clogP** and **LipE** values was selected to generate a pharmacophore model.

### 4.4. Pharmacophore Model Generation and Validation

To build a pharmacophore hypothesis to elucidate the 3D structural features of IP_3_R modulators, a ligand-based pharmacophore model was generated using LigandScout 4.4.5 software [126,127]. For ligand-based pharmacophore modeling, the 500 structural conformers of the template molecule were generated using an *iCon* setting [128] with a 0.7 root mean square (RMS) threshold. Then, clustering of the generated conformers was performed by using the radial distribution function (RDF) code algorithm [52] as a similarity measure [129]. The conformation value was set as 10 and the similarity value to 0.4, which is calculated by the average cluster distance calculation method [127]. To identify pharmacophoric features present in the template molecule and screening dataset, the Relative Pharmacophore Fit scoring function [54] was used. The Shared Feature option was turned on to score the matching features present in each ligand of the screening dataset. Excluded volumes from clustered ligands of the training set were generated, and the feature tolerance scale factor was set to 1.0. Default values were used for other parameters, and 10 pharmacophore models were generated for comparison and final selection of the IP_3_R-binding hypothesis.

The model with the best ligand scout score was selected for further analysis. To validate the pharmacophore model, the true positive (**TPR**) and true negative (**TNR**) prediction rates were calculated by screening each model against the dataset’s docked conformations. In LigandScout, the screening mode was set to ‘stop after first matching conformation’, and the Omitted Features option of the pharmacophore model was switched off. Additionally, pharmacophore-fit scores were calculated by the similarity index of hit compounds with the model. Overall, the model quality was accessed by applying Matthew’s correlation coefficient (**MCC**) to each model:

(3) $$MCC=\frac{TP*TN-FP*FN}{\sqrt{\left(TP+FP\right)\left(TP+FN\right)\left(TN+FP\right)\left(TN+FN\right)}}$$

The true positive rate (**TPR**) or sensitivity measure of each model was evaluated by applying the following equation:

(4) $$TPR=\frac{TP}{\left(TP+FN\right)}$$

Further, the true negative rate (**TNR**) or specificity (SPC) of each model was calculated by:

(5) $$TNR=\frac{TN}{\left(FP+TN\right)}$$

where true positives (**TP**) are active-predicted actives, and true negatives (**TN**) are inactive-predicted inactives. False positives (**FP**) are inactives, but predicted by the model as actives, while false negatives (**FN**) are actives predicted by the model as inactives.

### 4.5. Pharmacophore-Based Virtual Screening

To obtain new potential hits (antagonists) against IP_3_R, the ChemBridge database [60], NCI (National Cancer Institute) database (release 4) [61,62], and ZINC database [63] were virtually screened (VS) against the proposed final ligand-based pharmacophore model. To curate the datasets obtained from databases, several filters (i.e., fragments, molecules with MW < 200, and duplicate removal) were applied, and inconsistencies were removed. Afterward, the curated datasets were processed against five CYP filters (CYP 1A2, 2C9, 2C19, 2D6, and 3A4) by using an online chemical modeling environment (OCHEM) to obtain CYP non-inhibitors [65]. Furthermore for each CYP non-inhibitor, 1000 conformations were generated stochastically in MOE 2019.01 [66], and using a hERG filter [70], the hERG non-blockers were identified. Finally, the CYP non-inhibitors and hERG non-blockers were screened against our final pharmacophore model. The hits (antagonists) were further refined and shortlisted to identify compounds with exact feature matches.

Further, the prioritized hits (antagonists) were docked into an IP_3_R_3-binding_ pocket using induced fit docking protocol [118] in MOE version 2019.01 [66]. The same protocol used for the collected dataset of 40 ligands was used for docking new potential hits mentioned earlier in the Methods and Materials section, Molecular Docking Simulations. The final best docked poses were selected to compare the binding modes of newly identified hits with the template molecule by using protein–ligand interaction profiling (PLIF) analysis.

### 4.6. Grid-Independent Molecular Descriptor (GRIND) Calculation

GRIND variables are alignment-free molecular descriptors that are highly dependent upon 3D molecular conformations of the dataset [98,130]. To correlate the 3D structural features of IP_3_R modulators with their respective biological activity values, different three-dimensional molecular descriptors (GRIND) models were generated. Briefly, energy minimized conformations, standard 3D conformations generated by CORINA software [131], and induced fit docking (IFD) solutions were used as input to Pentacle software for the development of the GRIND model. A brief methodology of conformation generation protocol is provided in the supporting information.

GRIND descriptor computations were based upon the calculation of molecular interaction fields (MIFs) [132,133] by using different probes. Four different types of probes were used to calculate GRID-based fields as molecular interaction fields (MIFs), where Tip defined steric hot spots with molecular shape and Dry was specified for the hydrophobic contours. Additionally, hydrogen-bond interactions were represented by O and N1 probes, representing sp_2_ carbonyl oxygen defining the hydrogen-bond acceptor and amide nitrogen defining the hydrogen-bond donor probe, respectively [35]. Grid spacing was set as 0.5 Å (default value) while calculating MIFs. Molecular interaction field (MIF) calculations were performed by placing each probe at different GRID steps iteratively. Furthermore, total interaction energy (**E_xyz_**) as a sum of Lennard–Jones potential energy (**E_lj_**), electrostatic (**E_el_**) potential interactions, and hydrogen-bond (**E_hb_**) interactions was calculated at each grid point as shown in Equation (6) [134,135]:

(6) $$E_{xyz}=\sum E_{lj}+\sum E_{el}+\;\sum E_{hb}$$

The most significant MIFs calculated were selected by the AMANDA algorithm [136] for the discretization step based upon the distance and the intensity value of each node (ligand–protein complex) probe. Default energy cutoff values (−0.75, −0.5, −2.6, and −4.2 for Tip, Dry, O, and N1 probes, respectively) were used for the discretization of MIFs. The consistently large auto and cross-correlation (CLACC) [137] algorithm was used to encode the values of prefiltered (node–node) energy products into cross and auto correlogram (auto (Tip-Tip, Dry-Dry, O-O, N1-N1) and cross (Tip-Dry, Tip-O, Tip-N1, Dry-O, Dry-N1, O-N1)) GRIND variables. The leave-one-out (LOO) [78] procedure of the partial least square (PLS) analysis was used to correlate GRIND variables with the inhibitory potency (pIC_50_) values of the training set. The quality of the PLS model was accessed by the value of Q^2’^ and the standard deviation error of prediction (SDEP). To better understand how robust the final GRIND models were, the models were validated internally by correlating the GRIND variables with the inhibitory potency (pIC_50_) values of the test set. Furthermore, a fractional factorial design (FFD) variable selection algorithm was applied [76] to remove inconsistencies in GRIND variables and to improve the model statistics.

## 5. Conclusions

Despite the current therapies considering an optimal Ca^2+^ signaling role, pharmacological manipulation of IP_3_R-mediated Ca^2+^ signaling was proposed to improve antitumor treatments. For this purpose, our study demonstrated the important pharmacophoric features (a hydrogen-bond donor and acceptor group mapped from the hydrophobic group at a distance of 4.79 Å and 5.56 Å, respectively) of IP_3_R antagonists that may contribute to the effectiveness of the compounds in binding and inhibiting the IP_3_R-binding site. Moreover, some potential hits were identified against IP_3_R via virtual screening (VS) that may provide a solid basis for probing the IP_3_R inhibitors experimentally. Similarly, our GRIND model revealed the importance of a hydrophobic region that may define a molecular shape. The distances of complementary molecular features, such as hydrogen-bond donor and hydrogen-bond acceptor groups, were computed from the hydrophobic region at the virtual receptor site. The proposed 3D structural features of the IP_3_R virtual receptor site complementary with the pharmacophoric features of antagonists may provide an effective route for the synthesis of modulators in targeting the IP_3_R-binding site.

## Figures and Tables

**Table 1:** Ligand dataset of IP_3_R showing calculated log *p* values and LipE values.

Inositol Phosphate (IP) (Class A)										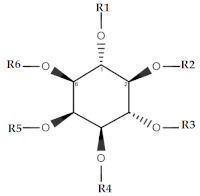					
Comp. No.	R_1_	R_2_	R_3_	R_4_	R_5_	R_6_	Conformation	Key Name		IC_50_ (µM)	logP	clogP	pIC_50_	LipE	Ref.
A_1_	PO_3_^−2^	PO_3_^−2^	OH	PO_3_^−2^	PO_3_^−2^	OH	R,S,S,S,S,S	DL-Ins(1,2,4,5)P4		0.03	−7.5	−7.2	1.6	14.8	[41]
A_2_	PO_3_^−2^	PO_3_^−2^	OH	PO_3_^−2^	PO_3_^−2^	OH	S,S,S,R,R,R	scyllo-Ins(1,2,4,5)P4		0.02	−7.5	−7.2	1.8	15.1	[42]
A_3_	PO_3_^−2^	PO_3_^−2^	OH	PO_3_^−2^	OH	OH	S,S,R,R,R,R	DL-scyllo-Ins(1,2,4)P3		0.05	−6.4	−5.7	1.3	13.1	[41]
A_4_	PO_3_^−2^	OH	PO_3_^−2^	PO_3_^−2^	PO_3_^−2^	OH	R,S,S,S,S,S	Ins(1,3,4,5)P4		0.01	−7.5	−6.5	2.5	15.1	[42]
A_5_	PO_3_^−2^	OH	PO_3_^−2^	PO_3_^−2^	OH	PO_3_^−2^	R,S,R,S,S,R	D-chiro-Ins(1,3,4,6)P4		0.17	−7.5	−6.7	0.7	13.4	[42]
A_6_	PO_3_^−2^	OH	OH	PO_3_^−2^	PO_3_^−2^	PO_3_^−2^	R,S,S,R,R,S	Ins(1,4,5,6)P4		0.43	−7.7	−8.5	0.2	14.9	[41]
A_7_	PO_3_^−2^	OH	OH	PO_3_^−2^	PO_3_^−2^	OH	R,R,S,R,R,S	Ins(1,4,5)P3		3.01	−6.4	−5.8	2.2	14.1	[42]
A_8_	PO_3_^−2^	OH	OH	OH	PO_3_^−2^	PO_3_^−2^	R,R,S,R,R,S	Ins(1,5,6)P3		0.04	−6.2	−5.8	0.4	13.1	[42]
A_9_	OH	OH	PO_3_^−2^	PO_3_^−2^	PO_3_^−2^	PO_3_^−2^	S,R,R,S,R,S	Ins(3,4,5,6)P4		0.62	−7.7	−7.2	1.3	13.4	[41]
A_10_	OH	OH	PO_3_^−2^	PO_3_^−2^	PO_3_^−2^	OH	S,S,R,R,S,S	Ins(3,4,5)P3		0.01	−6.6	−5.7	1.9	13.9	[41]
A_11_	OH	OH	OH	PO_3_^−2^	PO_3_^−2^	PO_3_^−2^	R,S,S,S,R,S	Ins(4,5,6)P3		93.0	−6.9	−5.8	−1.3	9.8	[43]
A_12_	OH	OH	OH	PO_3_^−2^	PO_3_^−2^	OH	R,R,S,S,R,S	Ins(4, 5)P2		20.0	−5.5	−4.3	−0.5	9.1	[43]
**Xestospongins (Xe)** **(Class B)**										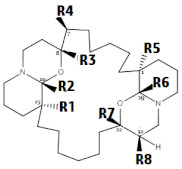					
**Comp. No.**	**R_1_**	**R_4_**	**R_5_**	**R_8_**	**Conformation**		**Key Name**		**IC_50_ (µM)**		**logP**	**clogP**	**pIC_50_**	**LipE**	**Ref.**
B_1_	OH	---	OH	---	R,R,S,R,R,S		Araguspongine C		6.60		5.7	4.7	5.2	0.5	[44]
B_2_	OH	---	---	CH_3_	S,S,R,S,R,R,R		Xestospongin B		5.01		6.8	7.2	5.3	−1.9	[45]
B_3_	OH	---	---	---	S,S,R,R,S,R		Demethylated Xestospongin B		5.86		6.5	6.8	5.2	−1.5	[46]
B_4_	---	OH	---	---	S,S,R,R,S,S,R		7-(OH)-XeA		6.40		6.3	6.8	5.2	−1.5	[44]
B_5_	---	---	---	---	S,S,R,S,S,R		Xestospongin A		2.53		7.3	8.1	5.6	−2.4	[46]
B_6_	---	---	---	---	R,S,R,R,S,R		Araguspongine B		0.65		7.3	8.0	6.2	−1.8	[46]
**Benzene Phosphate Derivatives** **(Class C)**										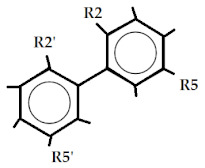					
**Comp. No.**	**R_2_**	**R_2′_**	**R_3′_**	**R_4_**	**R_4′_**	**R_5_**	**R_5′_**	**R_6_**	**Key Name**	**IC_50_ (µM)**	**logP**	**clogP**	**pIC_50_**	**LipE**	**Ref.**
C_1_	PO_3_^−2^	---	PO_3_^−2^	PO_3_^−2^	---	----	PO_3_^−2^	PO_3_^−2^	BiPh(2,3′,4,5′,6)P5	0.42	−1.2	−4.2	6.3	14.9	[47]
C_2_	PO_3_^−2^	PO_3_^−2^	---	PO_3_^−2^	PO_3_^−2^	PO_3_^−2^	PO_3_^−2^	---	BiPh(2,2′4,4′,5,5′)P6	0.19	−2.8	−6.1	6.7	17.2	[47]
C_3_	PO_3_^−2^	PO_3_^−2^	---	PO_3_^−2^	PO_3_^−2^	PO_3_^−2^	PO_3_^−2^	---	1,2,4-Dimer Biph(2,2′,4,4′,5,5′)P6	0.38	−3.9	−8.2	6.4	14.7	[47]

**Table 2:** The identified pharmacophoric features and mutual distances (A◦), along with ligand scout score and statistical evaluation parameters.

Model No.	Pharmacophore Model (Template)	Model Score	Model Distance						Model Statistics	
1.	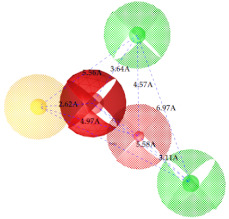	0.68 *		Hyd	HBA_1_	HBA_2_	HBD_1_	HBD_2_	TP: TN: FP: FN: **MCC:**	87% 72% 06% 03% **0.76**
			Hyd	0						
			HBA_1_	2.62	0					
			HBA_2_	4.79	2.61	0				
			HBD_1_	5.56	3.64	4.57	0			
			HBD_2_	7.68	5.58	3.11	6.97	0		
2.	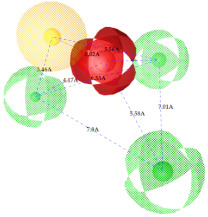	0.67		Hyd	HBA_1_	HBD_1_	HBD_2_	HBD_3_	TP: TN: FP: FN: MCC:	51% 70% 14% 18% 0.26
			Hyd	0						
			HBA_1_	2.48	0					
			HBD_1_	3.46	4.17	0				
			HBD_2_	5.56	3.63	6.33	0			
			HBD_3_	7.43	5.58	7.8	7.01	0		
3.	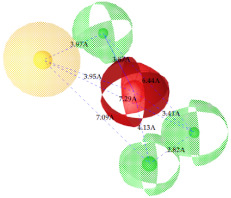	0.66		Hyd	HBA	HBD_1_	HBD_2_	HBD_3_	TP: TN: FP: FN: MCC:	72% 29% 12% 33% 0.02
			Hyd	0						
			HBA	3.95	0					
			HBD_1_	3.97	3.87	0				
			HBD_2_	7.09	4.13	2.86	0			
			HBD_3_	7.29	3.41	7.01	2.62	0		
4.	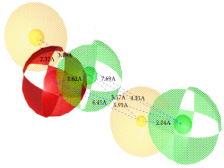	0.65		Hyd	HBA	HBD_1_	HBD_2_	Hyd	TP: TN: FP: FN: MCC:	49% 71% 14% 27% 0.23
			Hyd	0						
			HBA	2.32	0					
			HBD_1_	3.19	1.62	0				
			HBD_2_	7.69	6.91	4.57	0			
			Hyd	6.22	4.41	3.17	2.04	0		
5.	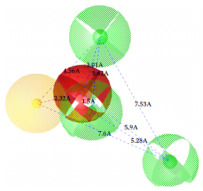	0.64		Hyd	HBA	HBD_1_	HBD_2_	HBD_3_	TP: TN: FP: FN: MCC:	54% 57% 28% 27% 0.13
			Hyd	0						
			HBA	2.32	0					
			HBD_1_	4.56	3.01	0				
			HBD_2_	2.92	1.05	3.61	0			
			HBD_3_	7.06	5.09	7.53	5.28	0		
6.	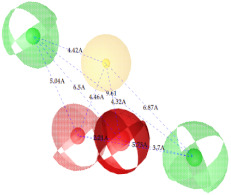	0.63		Hyd	HBA_1_	HBA_2_	HBD_1_	HBD_2_	TP: TN: FP: FN: MCC:	60% 29% 57% 45% −0.07
			Hyd	0						
			HBA_1_	4.32	0					
			HBA_2_	4.46	2.21	0				
			HBD_1_	6.87	3.07	5.73	0			
			HBD_2_	4.42	6.05	5.04	9.61	0		
7.	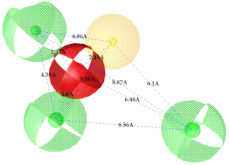	0.62		Hyd	HBA	HBD_1_	HBD_2_	HBD_3_	TP: TN: FP: FN: MCC:	63% 71% 14% 42% 0.32
			Hyd	0						
			HBA	2.49	0					
			HBD_1_	4.06	2.07	0				
			HBD_2_	5.08	2.8	2.38	0			
			HBD_3_	6.1	6.48	8.87	6.56	0		
8.	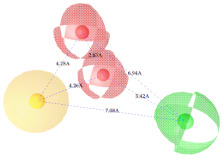	0.61		Hyd	HBA_1_	HBA_2_	HBD		TP: TN: FP: FN: MCC:	55% 57% 42% 48% 0.08
			Hyd	0						
			HBA_1_	4.28	0					
			HBA_2_	4.26	2.8	0				
			HBD	7.08	6.94	5.42	0			
					
9.	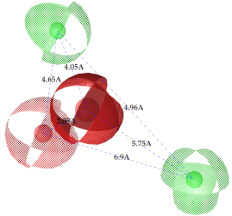	0.60		HBA_1_	HBA_2_	HBA_3_	HBD_1_	HBD_2_	TP: TN: FP: FN: MCC:	58% 28% 57% 48% −0.09
			HBA_1_	0						
			HBA_2_	2.52	0					
			HBA_3_	2.05	2.07	0				
			HBD_1_	4.65	2.28	4.06	0			
			HBD_2_	6.9	7.96	5.75	8.96	0		
10.	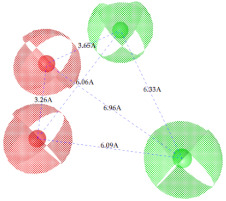	0.60		HBA_1_	HBA_2_	HBD_1_	HBD_2_		TP: TN: FP: FN: MCC:	51% 42% 40% 54% −0.01
			HBA_1_	0						
			HBA_2_	3.26	0					
			HBD_1_	3.65	6.06	0				
			HBD_2_	6.96	6.09	6.33	0			
					

Where, Hyd = Hydrophobic, HBA = Hydrogen bond acceptor, HBD = Hydrogen bond donor, TP = True positives, TN = True negatives, FP = False positives, FN = False negatives and MCC = Matthew’s correlation coefficient. * Finally selected model based upon ligand scout score, sensitivity, specificity, and Matthew’s correlation coefficient.

**Table 3:** Summarizing the statistical parameters of independent partial least square (PLS) models generated by using different 3D conformational inputs in GRIND.

Conformational Method	Fractional Factorial Design (FFD) Cycle									Comments FFD_2_ (LV_2_)
	Complete			FFD_1_			FFD_2_			
	Q^2^_LOO_	R^2^	SDEP	Q^2^_LOO_	R^2^	SDEP	Q^2^_LOO_	R^2^	SDEP	
Energy Minimized	0.07	0.93	2.8	0.12	0.93	2.7	0.23	0.94	2.5	Inconsistent for auto- and cross-GRID variables
Standard 3D	0.59	0.68	3.5	0.15	0.56	3.5	0.05	0.53	3.5	Inconsistent for auto- and cross-GRID variables
Induced Fit Docked	0.61	0.64	1.1	0.68	0.71	1.0	*0.70	**0.72**	**0.9**	Consistent for Dry-Dry, Dry-O, Dry-N1, and Dry-Tip correlogram (Figure 3)

* Bold values show the statistics of the final selected model.

**Table 4:** The pairwise comparison of the ligand-based pharmacophore features with their complementary GRIND model features representing the virtual receptor site (VRS).

Pharmacophore (Ligand-Based)		GRIND (Correlogram)		
Pharmacophore Variables	Distances	GRIND Variables	Features at VRS	Distance
Hydro-HBA Hydro-HBD HBD-HBD	4.79 Å 5.56 Å 6.97 Å	Dry-N1 Dry-O O-O	Hyd-HBD Hyd-HBA HBA-HBA	7.6–8 Å 6.8–7.2 Å 10.4–10.8 Å

## Data Availability

Not applicable.

## Supplementary Materials

The following are available online at https://www.mdpi.com/article/10.3390/ijms222312993/s1. References [1,2,3,4,5,6,7,8,9,10,11,12,13,14,15,16,17,18,19,20,21,22,23] are cited in the Supplementary Materials.
