# Supplementary material for: Combined Pharmacophore and Grid-Independent Molecular Descriptors (GRIND) Analysis to Probe 3D Features of Inositol 1,4,5-Trisphosphate Receptor (IP3R) Inhibitors in Cancer
Source: Int J Mol Sci. 2021 Nov 30;22(23):12993. doi: 10.3390/ijms222312993 (PMC8657927; doi:10.3390/ijms222312993)
Supplement: Supplementary file 1 [file ijms-22-12993-s001.zip › ijms-1405565-supplementary-R3 (1).pdf]

International Journal of *Molecular Sciences*

MDPI

Supporting Information

Combined Pharmacophore and Grid Independent Molecular Descriptors (GRIND) Analysis to Probe 3D features of Inositol 1, 4, 5-trisphosphate Receptor (IP_3R) Inhibitors in Cancer

Humaira Ismatullah and Ishrat Jabeen *

Research Centre for Modelling and Simulation (RCMS), NUST Interdisciplinary Cluster for Higher Education (NICHE), National University of Sciences and Technology (NUST), H-12 Islamabad (44000), Pakistan Email: Humaira (hismatullah.phd15@rcms.nust.edu.pk), Ishrat Jabeen (ishrat.jabeen@rcms.nust.edu.pk)

* Correspondence: Dr. Ishrat Jabeen, Associate Professor (HOD), Department of Computational Sciences RCMS, National University of Sciences and Technology (NUST)
Email: (ishrat.jabeen@rcms.nust.edu.pk); Tel.: +92-51-9085-5732

Supporting Information

Contents

1. Results

Table S1	The activity landscape and LipE profile of the IP_3R ligand dataset.
Figure S1	The physicochemical properties of the IP_3R ligand dataset.
Figure S2	A plot of pIC_{50} versus clogP showing Lipophilic Efficiency (LipE) Profile of IP_3R inhibitors.
Figure S3	Representation of chemical features by the pharmacophore model for the activity of ryanodine.
Figure S4	ROC curve between true positive rates (sensitivity) vs. false positive rate (1-specificity).
Figure S5	The compounds (2D structures) used as external test set to validate the pharmacophore model.
Figure S6	Chemical structures of Potential hits (IP_3R modulators)
Figure S7	A step by step protocol of the ligand based virtual screening
Figure S8	Docking solutions of proposed hits in IP_3R binding pocket.
Figure S9	A PCA plot between the first two principal components (PC) defining the descriptor space of the training set and test set.
Figure S10	Correlations plot between experimental versus predicted inhibitory potencies (pIC_{50}) of IP_3R antagonists by GRIND.
Table S2	The statistical parameters of cross-validation (Leave-Five-Out) of PLS models generated by GRIND.
Table S3	Experimental and predicted biological activity values (pIC_{50}) of training and test sets obtained after Leave-One-Out (LOO) cross-validation.
Table S4	The experimental inhibitory potency of test set and the modified r^2 (rm^2) calculated.
Figure S11	TIP contour around the template molecule showing the curved molecular boundary.
Figure S12	The binding pose of the template molecule representing important pharmacophoric features in complementing with amino acid residues within the IP_3R binding core

2. Materials and Methods

Figure S13	Step by step data curation process to obtain master database.
Figure S14	A correlation plot between binding energies of top docked poses vs. potential inhibitory potency (pIC_{50})
	Conformation generation protocol

1. Results

Table S1. The activity landscape and LipE profile of the IP_3R ligand dataset.

Comp	IC_{50}(μM)	logP(o/w)	cLogp	pIC50	LipE	Ref
A2	0.02	-7.5	-7.2	1.8	15.1	[1]
A8	0.04	-6.2	-5.8	0.4	13.1	[1]
A10	0.01	-6.6	-5.7	1.9	13.9	[2]
A6	0.43	-7.7	-8.5	0.2	14.9	[2]
M2	0.02	-4.8	-7.2	7.5	17.5	[3]
A1	0.03	-7.5	-7.2	1.6	14.8	[2]
A9	0.62	-7.7	-7.2	1.3	13.4	[2]
A7	3.01	-6.4	-5.8	2.2	14.1	[1]
**M19**	**0.05**	**1.5**	**2.71**	**6.7**	**4.5**	[4]
A5	0.17	-7.5	-6.7	0.7	13.4	[1]
C2	0.19	-2.8	-6.1	6.7	17.2	[5]
C3	0.38	-3.9	-8.2	6.4	14.7	[5]
C1	0.42	-1.2	-4.2	6.3	14.9	[5]
A3	0.05	0.05	0.05	0.05	0.05	[2]
A4	-6.4	-6.4	-6.4	-6.4	-6.4	[1]
B3	5.86	6.5	6.8	5.2	-1.5	[6]
B1	6.60	5.7	4.7	5.2	0.5	[7]
B5	2.53	7.3	8.1	5.6	-2.4	[6]
M4	5.00	-0.6	1.9	5.3	3.3	[8]
M3	5.00	1.5	7.2	5.3	-1.9	[9]
B6	0.65	7.3	8.0	6.2	-1.8	[6]
B2	5.01	6.8	7.2	5.3	-1.9	[10]
B4	6.40	6.3	6.8	5.2	-1.5	[7]
M5	15.0	2.3	1.6	4.8	3.2	[11]
A12	20.0	-5.5	-4.3	-0.5	9.1	[12]
M13	22.0	6.1	5.6	4.6	-0.9	[13]
M1	34.0	-2.9	-5.4	4.4	9.9	[14]
M7	50.0	2.2	1.9	4.3	2.4	[8]
M6	50.0	1.9	4.2	4.3	0.1	[15]
A11	93.0	-6.9	-5.8	-1.3	9.8	[12]
M9	120	0.9	3.5	3.9	0.3	[16]
M14	140	3.1	3.5	3.8	0.3	[13]
M15	145	4.6	4.3	3.8	-0.5	[13]
M16	160	3.1	4.0	3.7	-0.2	[13]
M8	340	1.1	2.3	3.4	1.1	[16]
M17	450	5.3	4.2	3.3	0.9	[13]
M18	2000	1.7	2.8	2.6	-0.1	[4]
M10	3300	-2.5	-2.9	2.4	5.4	[16]
M11	8700	-1.9	-1.9	2.0	3.9	[16]
M12	20000	-2.3	-2.0	1.6	3.7	[16]

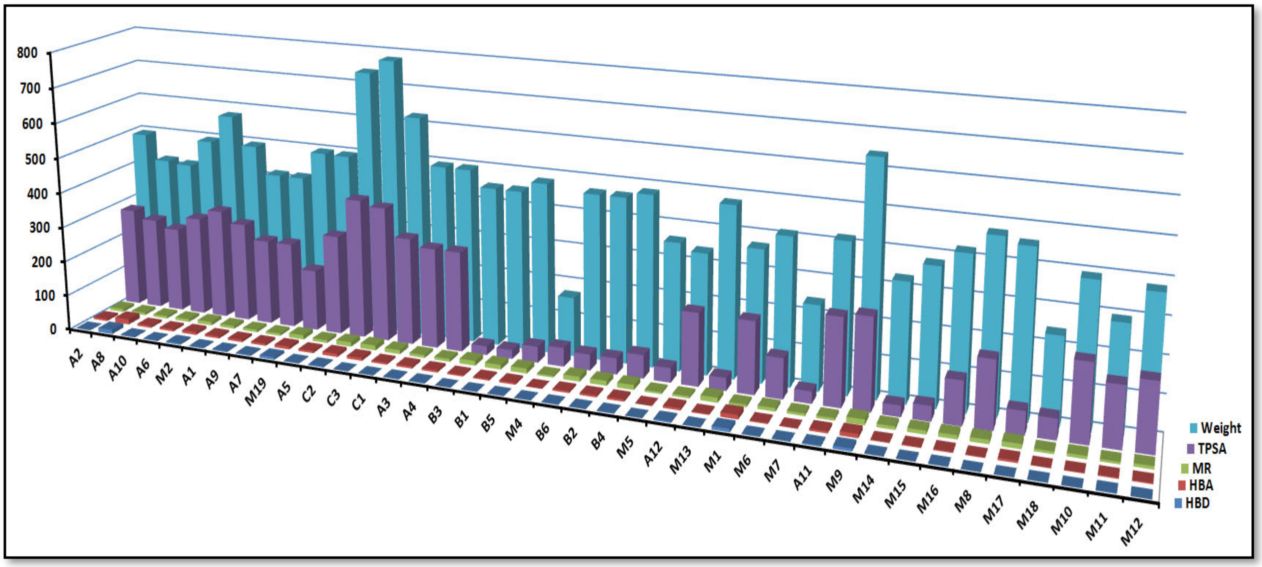

Figure S1. The physicochemical (molecular weight, TPSA, molar refractivity, and hydrogen bond acceptor/donor) properties of the IP_3R ligand dataset.

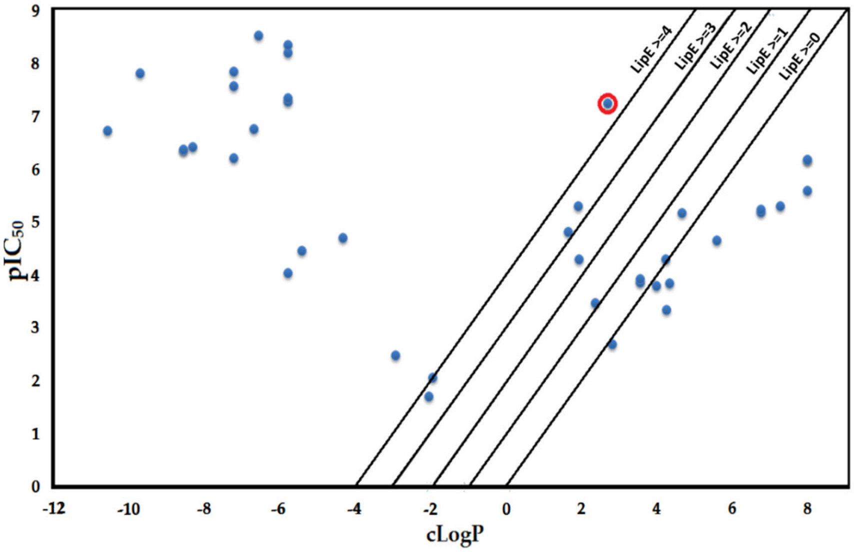

Figure S2. A plot of pIC_{50} (inhibitory Potency) versus clogP showing Lipophilic Efficiency (LipE) profile of IP_3R inhibitors. M_{19} (Ryanodine) is circled red and selected as a template molecule because of the lipophilic efficiency profile (the most potent compound in the dataset (IC_{50}: 0.055 μM) with a clogP value of 2.71 and LipE value of 4.6).

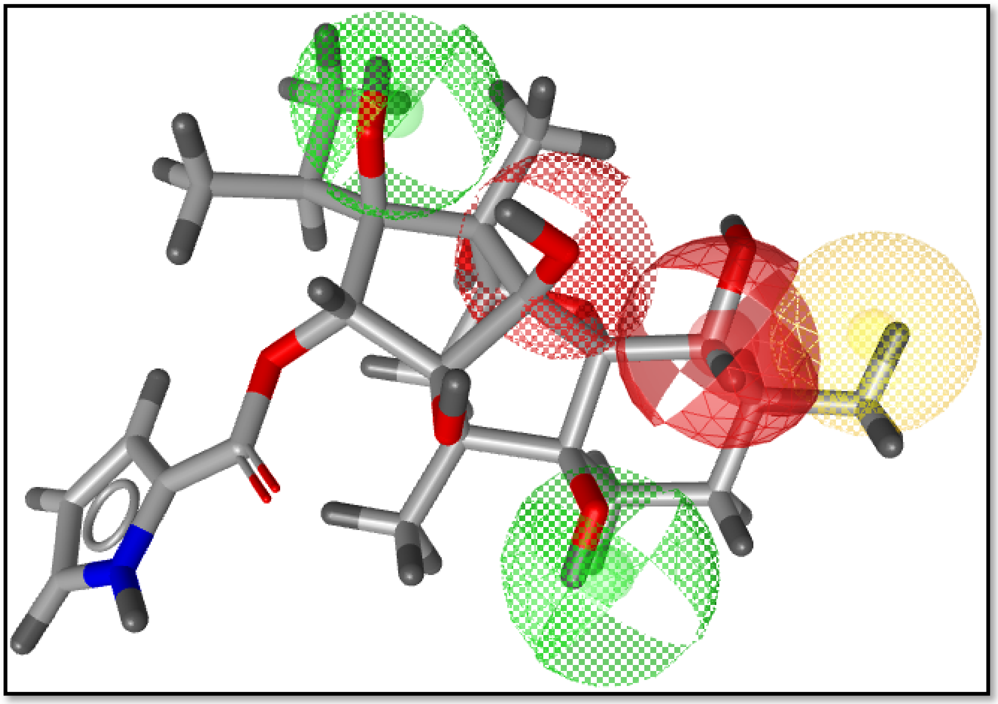

Figure S3: Shows the chemical features of the pharmacophore model responsible for the activity of ryanodine. The yellow circle represents the hydrophobic region. The hydrogen bond acceptor and hydrogen bond donors are represented by red and green circles respectively.

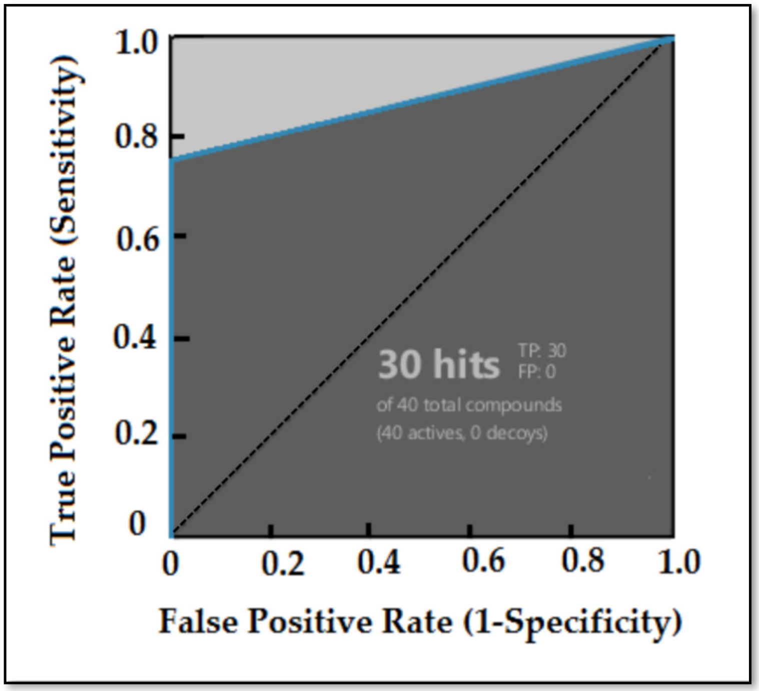

Figure S4. ROC curve between true positive (TP) rates (sensitivity) vs. false positive rate (1-specificity) of final selected pharmacophore model**.** Overall, out of 33 active compounds 30 were predicted as actives (TP) and 3 were predicted as inactive (FN).

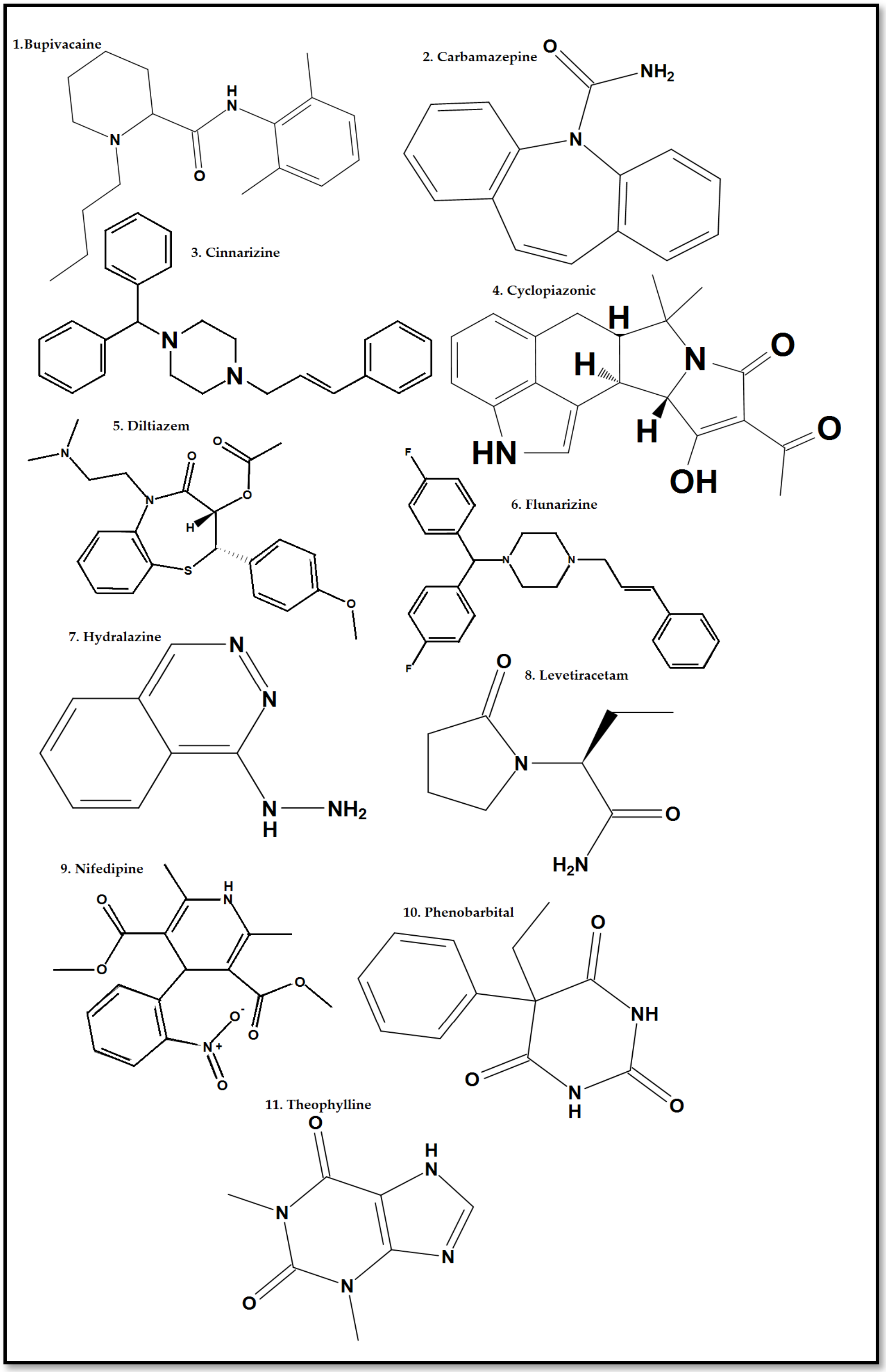

Figure S5. The chemical structures of the compounds of the external test set used to validate the pharmacophore model. This test set is named as 'Blind set', as the IC_{50} values were not defined in the literature [17-19].

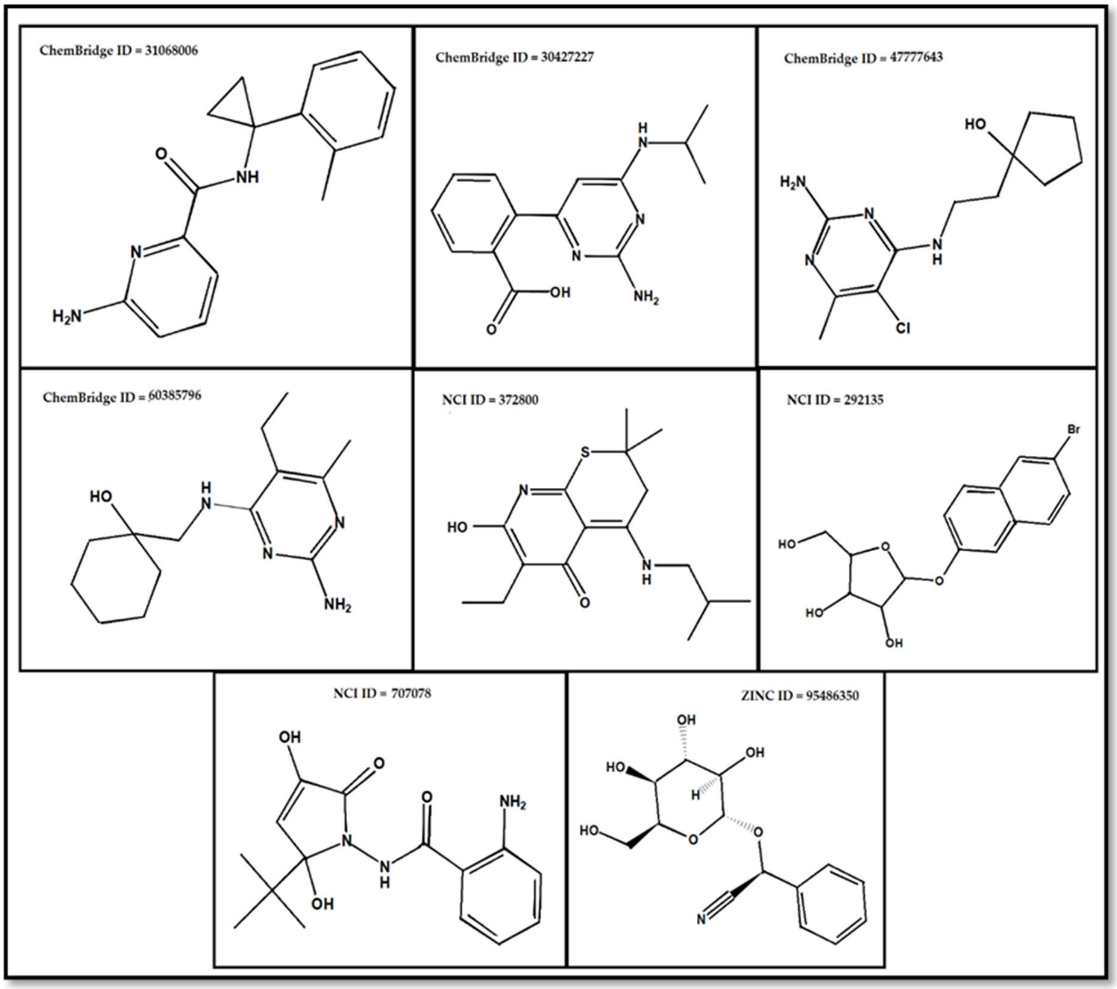

Figure S6. The chemical (2D) structures of the potential hit compounds shortlisted after pharmacophore based virtual screening of National Cancer Institute (NCI) database, ZINC database, and ChemBridge database.

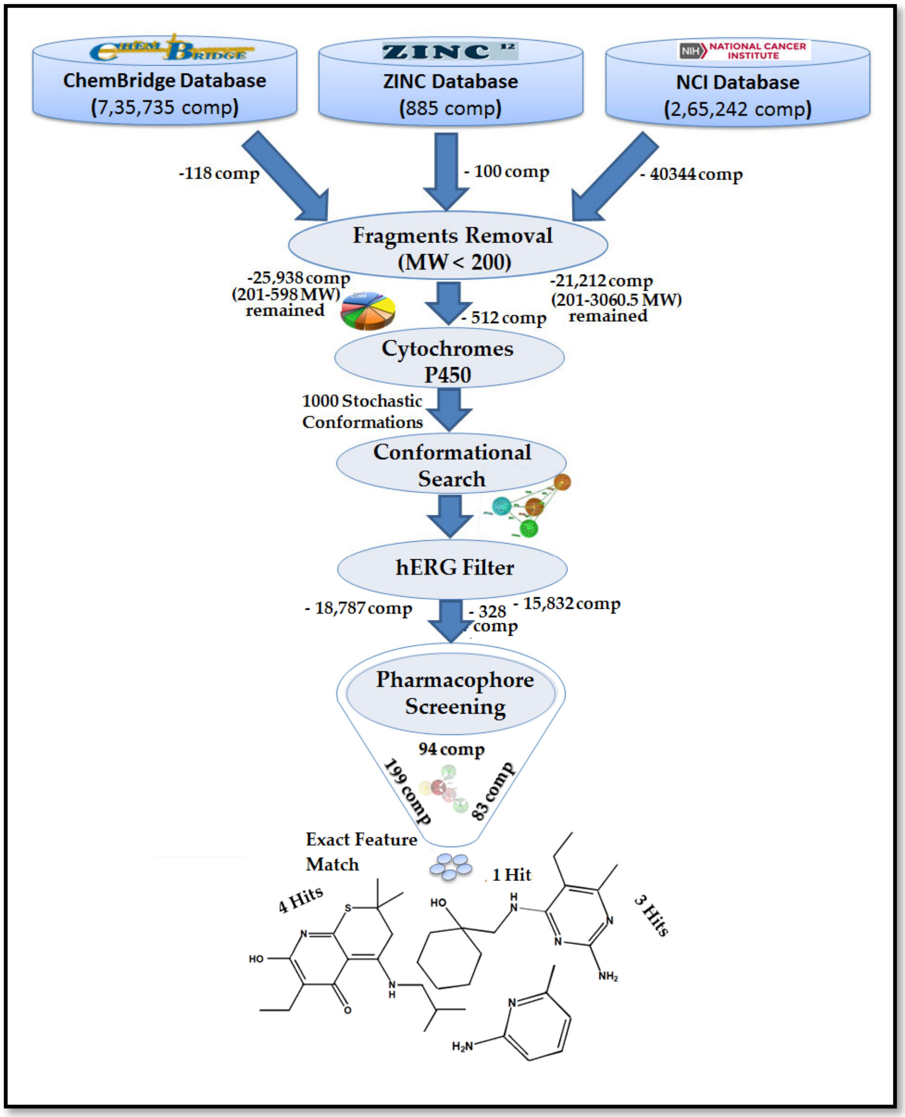

Figure S7. A step by step protocol of the ligand based virtual screening. The 735735 compounds from ChemBridge database, 885 (natural) compounds from Zinc database, and 265242 compounds from the National Cancer Institute (NCI) database were screened. After several filters application and Pharmacophore model screening the 4 hits from ChemBridge, 4 hits from NCI and 2 hits from Zinc database were shortlisted as IP_3R modulators (antagonists).

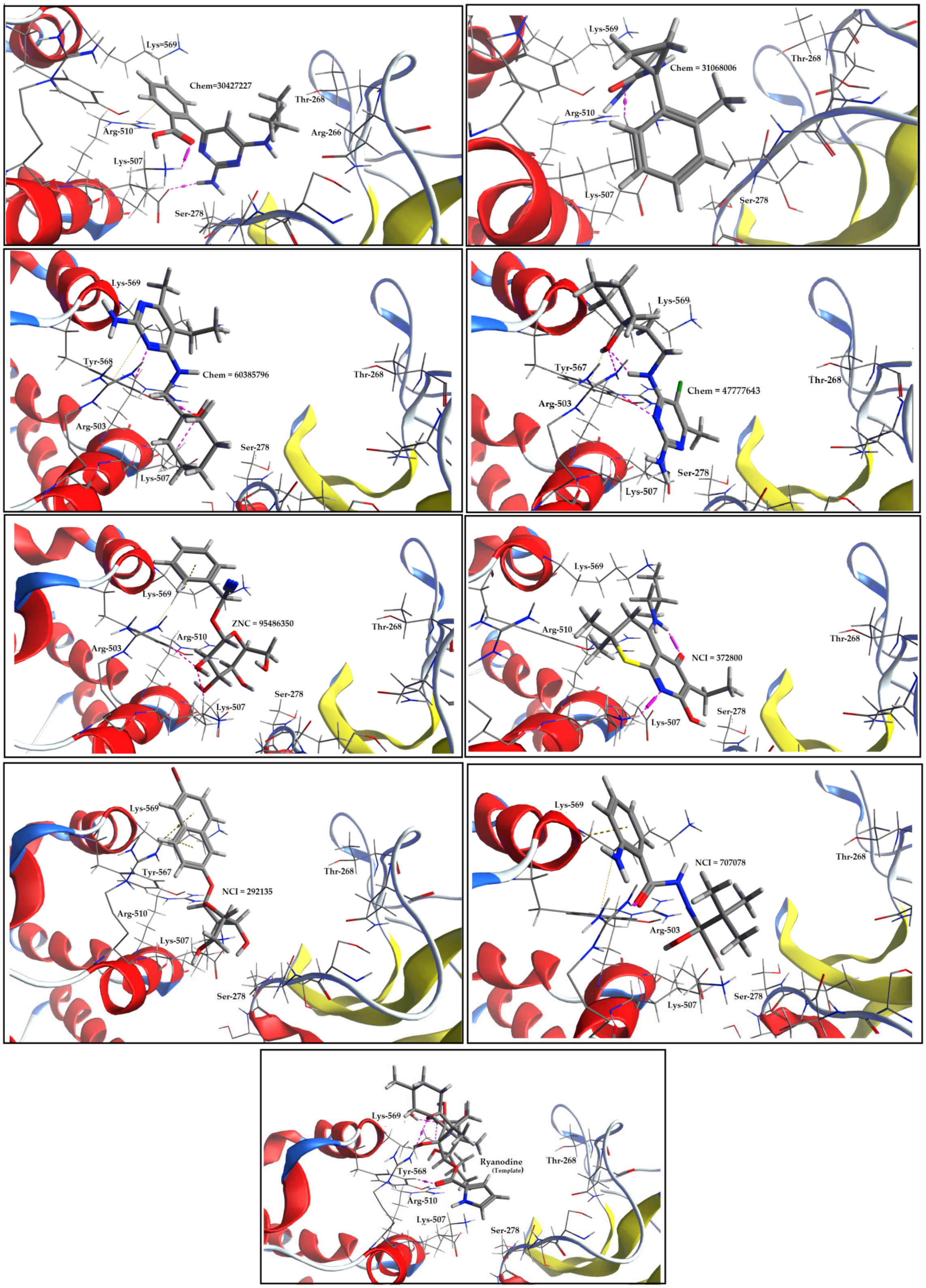

Figure S8. The best docked poses of shortlisted hits within the binding pocked of protein. The IP_3R_3 protein is shown in backbone secondary structure and the interacting residues are shown in stick representation. Mostly, the ligand molecules interacted with Lys-569 and Lys-507 forming a π-π interaction or surface contacts. Ligands interacted with Arg-503 and Arg-510 via hydrogen bond acceptor and donor interactions.

1. **Principal Component Analysis:**

The principal component analysis (PCA) [20] was performed to determine the structural variance in the training dataset by computing the complete GRIND descriptors set. In the training data set, 40 % structural variance has been described by the first two principal components (PC1 & PC2) (figure S9). The compounds in the form of a cluster at the right bottom side showed the molecules with a small structure containing only one hydrophobic ring, mainly class 'A' compounds and some from the class 'M'. Overall, the other compounds are more diverse with elongated chemical scaffolds and larger molecular weight, describing the data diversity.

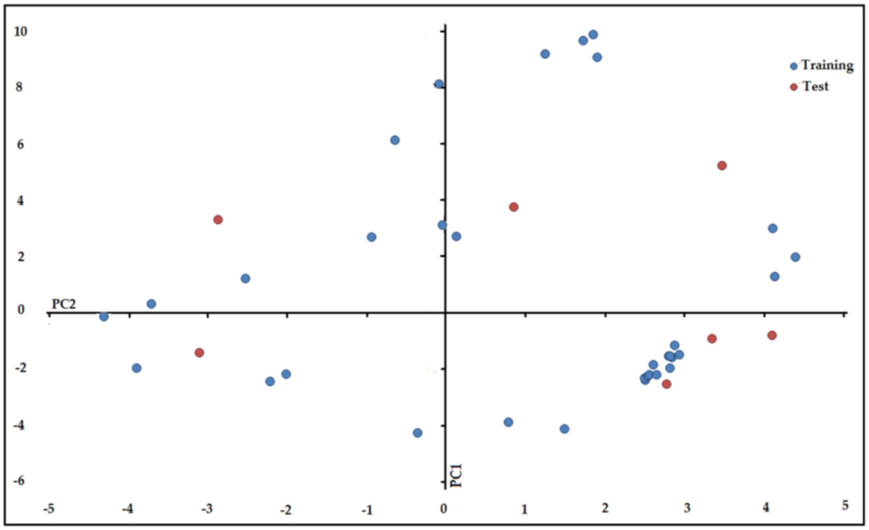

Figure S9. A PCA plot between the first two principal components (PC1 & PC2) defining the descriptor space of the training set and test set.

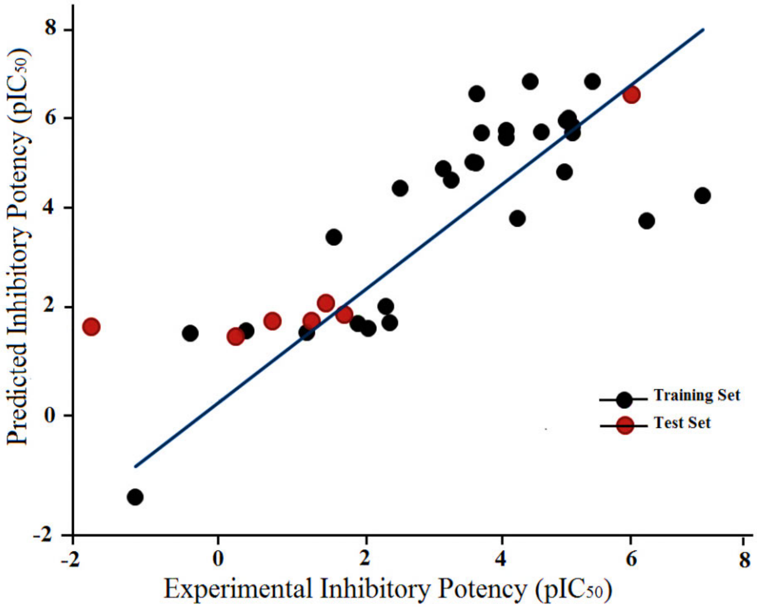

Figure S10. Representing a correlation plot between experimental versus predicted inhibitory potencies (pIC_{50}) of IP_3R antagonists. The training set is represented by black circles while the test set is represented by red circles.

Table S2. The statistical parameters of PLS models generated by GRIND measured by cross-validation method.

Cross-Validation Method	Fractional Factorial Design (FFD) Cycle								
	Complete			FFD_1			FFD_2		
	Q^2	R^2	SDEP	Q^2	R^2	SDEP	Q^2	R^2	SDEP
LOO	0.61	0.64	1.1	0.68	0.71	1.0	**0.70**	**0.72**	**0.9**
LFO (1-5)	0.60	0.62	1.2	0.62	0.63	1.1	**0.64**	**0.66**	**1.0**
LFO (5-10)	0.62	0.64	1.1	0.64	0.65	1.0	**0.62**	**0.65**	**1.1**
LFO (10-15)	0.59	0.61	1.3	0.59	0.60	1.2	**0.59**	**0.61**	**1.3**
LFO (16-20)	0.58	0.60	1.2	0.59	0.61	1.1	**0.60**	**0.61**	**1.1**
LFO (21-25)	0.59	0.62	1.4	0.58	0.61	1.3	**0.60**	**0.62**	**1.2**
LFO (26-30)	0.61	0.63	1.2	0.61	0.60	1.3	**0.60**	**0.61**	**1.3**

LOO = Leave-One-Out, LFO = Leave-Five-Out

Table S3. The experimental inhibitory potency compared with inhibitory potency predicted by GRIND. The residual values of ± 2 log units are considered optimal.

Comp	Experimental pIC_{50}	Predicted pIC_{50}	Residual Value	rm^2	S_{new}	AD (Outlier)	Comp	Experimental pIC_{50}	Predicted pIC_{50}	Residual Value	rm^2	S_{new}	AD (Outlier)
Training Set							Training Set						
A_4	2.53	1.24	1.28	-0.33	1.29	-	M_6	4.3	4.36	-0.06	3.24	2.59	-
A_8	0.36	1.12	-0.76	0.46	0.96	-	M_7	4.3	4.24	0.05	3.33	0.8	-
A_3	1.28	1.09	0.18	0.73	2.61	-	A_1	1.57	1.56	0.01	1.43	1.12	-
A_{12}	-0.47	1.08	-1.55	0.11	0.93	-	M_9	3.92	4.32	-0.4	1.44	1.52	-
A_7	2.21	1.16	1.05	-0.05	0.96	-	C_2	6.7	4.9	1.8	-2.28	1.18	-
A_{11}	-1.3	-1.56	0.26	-0.63	0.59	-	A_5	0.76	1.28	-0.52	0.21	1.69	-
B_5	5.59	5.15	0.43	1.92	1.21	-	A_6	0.2	1.03	-0.82	0.01	1.31	-
B_4	5.19	4.51	0.67	0.94	1.1	-	C_1	6.3	4.46	1.84	-2.24	1.61	-
B_1	5.18	3.69	1.48	-1.12	1.13	-	M_{13}	4.65	5.15	-0.49	1.39	1.53	-
B_2	5.3	4.43	0.86	0.38	1.15	-	M_{14}	3.85	4.95	-1.1	-0.18	1.47	-
B_3	5.23	4.56	0.66	0.98	1.34	-	M_{15}	3.83	3.84	-0.01	3.44	0.79	-
C_3	6.42	2.9	3.51	-5.6	1.58	-	M_{16}	3.79	3.85	-0.06	2.86	0.89	-
M_1	4.46	2.93	1.53	-1.05	1.04	-	A_2	1.85	1.38	0.46	0.59	1.19	-
M_4	5.3	4.32	0.97	0.08	2.49	-	A_9	1.35	1.28	0.07	0.99	1.07	-
M_5	4.82	4.33	0.48	1.48	1.82	-	M_{19}	7.25	5.53	1.72	-2.25	1.69	-

Table S4. The experimental inhibitory potency of test set compared with inhibitory potency predicted by GRIND. The modified r^2 (rm^2) calculated and the values greater than 0.5 are considered optimal.

Comp	Experimental pIC_{50}	Predicted pIC_{50}	Residual Value	rm^2	S_{new}	AD_Info (Outlier)
**Test Set**						
M_8	3.46	3.55	-0.09	2.434	1.29	-
M_{11}	2.06	1.24	0.81	0.195	1.13	-
M_{12}	1.69	2.63	-0.93	0.576	1.16	-
M_{10}	2.48	1.51	0.96	0.464	1.72	-
M_{17}	3.34	3.74	-0.4	1.222	1.42	-
M_{18}	2.69	3.42	-0.72	0.393	0.92	-
A_{10}	1.96	1.18	0.78	0.596	0.54	-
B_6	6.18	4.94	1.23	0.966	2.29	-

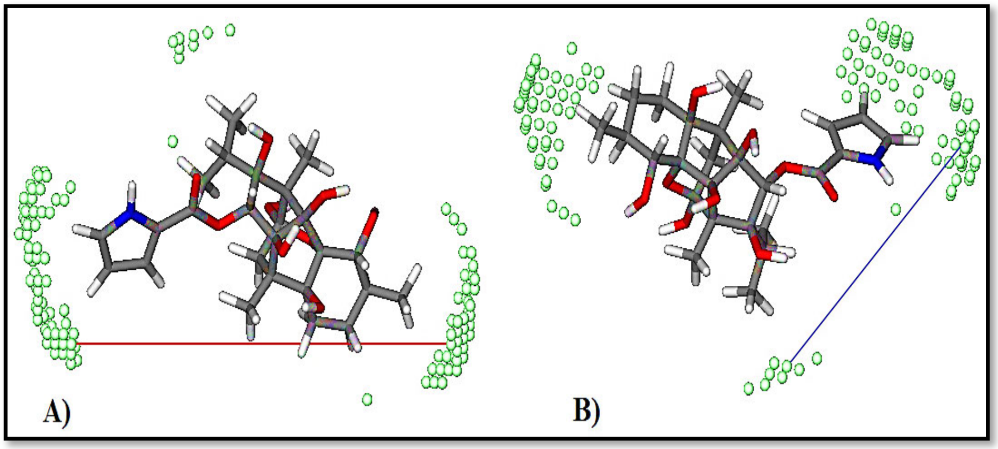

Figure S11. (**A**). TIP contour around the template molecule showing the curved molecular boundary at a wider distance of 16.40 Å - 16.80Å is positively correlated with the inhibitory potency of IP_3R. (**B**). whereas, the linear formed TIP at a shorter distance of 10.00 Å - 10.40Å is negatively correlated with the inhibitory potency of IP_3R.

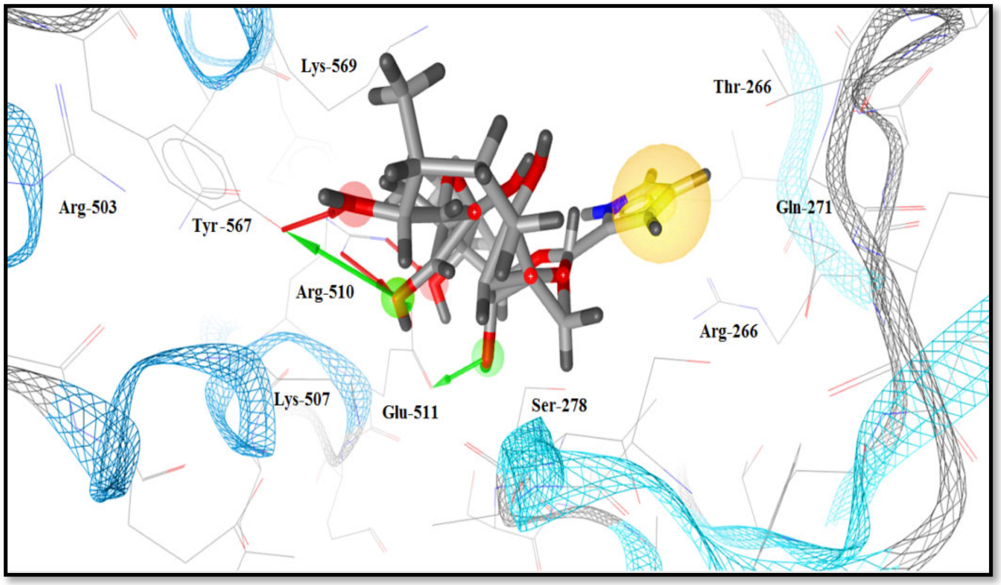

Figure S12. The binding pose of template molecule representing important pharmacophoric features in complementing with amino acid residues within IP_3R binding core.

2. Materials and Methods

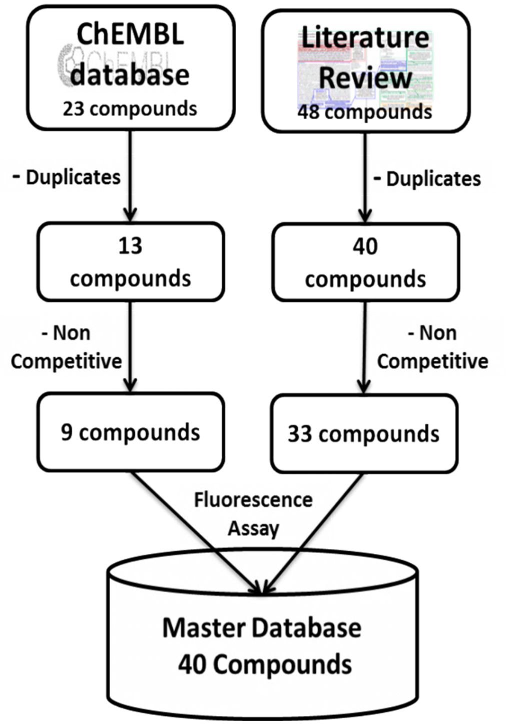

Figure S13. Step-by-step data curation process to obtain a master database of IP_3R.

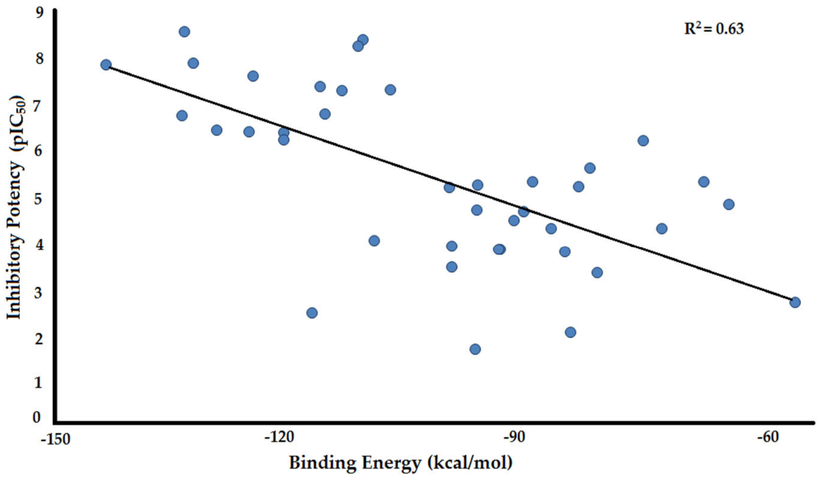

Figure S14. A correlation plot between binding energies of top docked poses vs. potential inhibitory potency (pIC_{50}) showing good correlation i.e. 0.63.

2. Conformational Analysis of Ligand dataset for GRIND:

1. **Energy minimized conformations**

Briefly, a stochastic search algorithm in MOE 2019.01 [21] was applied to generate energy minimized conformations of the ligand dataset. The generated conformations were ranked according to their energy values and a total of 300 conformations were produced. Each ligand with the lowest energy score was considered for the GRIND analysis.

2. **Standard 3D conformations**

To obtain standard 3D conformations of the ligand dataset, an online version of CORINA software [22] was used. The 3D model of a molecule is built-in CORINA by connecting the mono-centric fragments with standard bond lengths and bond angles. The dihedral angles along with torsion angles of ring systems and the Van der Waals and electrostatic (non-bonded) interactions are also considered and minimized. The final 3D conformation of each ligand was further subjected to Pentacle v 1.0.7 [23] as input for GRIND analysis.

3. References

1. Adelt, S., et al., *Enzyme-assisted total synthesis of the optical antipodes d-myo-inositol 3, 4, 5-trisphosphate and d-myo-inositol 1, 5, 6-trisphosphate: aspects of their structure– activity relationship to biologically active inositol phosphates.* Journal of medicinal chemistry, 1999. **42**(7): p. 1262-1273.
2. Liu, C., et al., *Synthesis, Calcium Mobilizing, and Physicochemical Properties of d-c hiro-Inositol 1, 3, 4, 6-Tetrakisphosphate, a Novel and Potent Ligand at the d-m yo-Inositol 1, 4, 5-Trisphosphate Receptor.* Journal of medicinal chemistry, 1999. **42**(11): p. 1991-1998.
3. Hill, T.D., P.-O. Berggren, and A.L. Boynton, *Heparin inhibits inositol trisphosphate-induced calcium release from permeabilized rat liver cells.* Biochemical and biophysical research communications, 1987. **149**(3): p. 897-901.
4. Bai, Y., M. Edelmann, and M.J. Sanderson, *The contribution of inositol 1, 4, 5-trisphosphate and ryanodine receptors to agonist-induced Ca2+ signaling of airway smooth muscle cells.* American Journal of Physiology-Lung Cellular and Molecular Physiology, 2009. **297**(2): p. L347-L361.
5. Mills, S.J., et al., *Multivalent benzene polyphosphate derivatives are non-Ca2+-mobilizing Ins (1, 4, 5) P3 receptor antagonists.* Messenger, 2012. **1**(2): p. 167-181.
6. Gafni, J., et al., *Xestospongins: potent membrane permeable blockers of the inositol 1, 4, 5-trisphosphate receptor.* Neuron, 1997. **19**(3): p. 723-733.
7. Ta, T.A., et al., *Hydroxylated xestospongins block IP3-induced Ca2+ release and sensitize Ca2+-induced Ca2+ release mediated by ryanodine receptors.* Molecular Pharmacology, 2005.
8. Palade, P., et al., *Pharmacologic differentiation between inositol-1, 4, 5-trisphosphate-induced Ca2+ release and Ca2+-or caffeine-induced Ca2+ release from intracellular membrane systems.* Molecular Pharmacology, 1989. **36**(4): p. 673-680.
9. Föhr, K., et al., *Decavanadate displaces inositol 1, 4, 5-trisphosphate (IP3) from its receptor and inhibits IP3 induced Ca2+ release in permeabilized pancreatic acinar cells.* Cell calcium, 1991. **12**(10): p. 735-742.
10. Jaimovich Pérez, E., et al., *Xestospongin B, a competitive inhibitor of IP3-mediated Ca2+ signalling in cultured rat myotubes, isolated myonuclei, and neuroblastoma (NG108-15) cells.* 2005.
11. Adunyah, S.E. and W. Dean, *Effects of sulfhydryl reagents and other inhibitors on Ca2+ transport and inositol trisphosphate-induced Ca2+ release from human platelet membranes.* Journal of Biological Chemistry, 1986. **261**(28): p. 13071-13075.
12. Podeschwa, M., et al., *Stereoselective Synthesis of myo-, neo-, L-chiro, D-chiro, allo-, scyllo-, and epi-Inositol Systems via Conduritols Prepared from p-Benzoquinone.* European Journal of Organic Chemistry, 2003. **2003**(10): p. 1958-1972.
13. Sczekan, S.R. and F. Strumwasser, *Antipsychotic drugs block IP3-dependent Ca2+-release from rat brain microsomes.* Biological psychiatry, 1996. **40**(6): p. 497-502.
14. Poitras, M., et al., *Interaction of benzene 1, 2, 4-trisphosphate with inositol 1, 4, 5-trisphosphate receptor and metabolizing enzymes.* European Journal of Pharmacology: Molecular Pharmacology, 1993. **244**(3): p. 203-210.
15. Akl, H., et al., *HA14-1, but not the BH3 mimetic ABT-737, causes Ca2+ dysregulation in platelets and human cell lines.* Haematologica, 2013: p. haematol. 2012.080598.
16. Richardson, A. and C.W. Taylor, *Effects of Ca2+ chelators on purified inositol 1, 4, 5-trisphosphate (InsP3) receptors and InsP3-stimulated Ca2+ mobilization.* Journal of Biological Chemistry, 1993. **268**(16): p. 11528-11533.
17. Seiler, S.M., A.J. Arnold, and H.C. Stanton, *Inhibitors of inositol trisphosphate-induced Ca2+ release from isolated platelet membrane vesicles.* Biochemical pharmacology, 1987. **36**(20): p. 3331-3337.
18. Nagarkatti, N., L.S. Deshpande, and R.J. DeLorenzo, *Levetiracetam inhibits both ryanodine and IP3 receptor activated calcium induced calcium release in hippocampal neurons in culture.* Neuroscience letters, 2008. **436**(3): p. 289-293.
19. Gurney, A.M. and M. Allam, *Inhibition of calcium release from the sarcoplasmic reticulum of rabbit aorta by hydralazine.* British journal of pharmacology, 1995. **114**(1): p. 238-244.
20. Wold, S., et al., *Multi-way principal components-and PLS-analysis.* Journal of chemometrics, 1987. **1**(1): p. 41-56.
21. *Molecular Operating Environment (MOE),*, Chemical Computing Group Inc.,2019.01., 1010 Sherbooke St. West, Suite #910, Montreal, QC, Canada, H3A 2R7, 2015.49.
22. Classic, C., *Performance improvements, new functionalities and applications of the 3D structure generator CORINA Classic.*
23. *Pentacle, Version 1.0.7; Molecular Discovery Ltd.: Perugia, Italy; 2009.*
